# KDM6A promotes imatinib resistance through YY1-mediated transcriptional upregulation of TRKA independently of its demethylase activity in chronic myelogenous leukemia

**DOI:** 10.7150/thno.50571

**Published:** 2021-01-01

**Authors:** Chengwan Zhang, Li Shen, Yifu Zhu, Ran Xu, Zhikui Deng, Xiaoning Liu, Yihan Ding, Chunling Wang, Yuye Shi, Liye Bei, Dongping Wei, Rick F. Thorne, Xu Dong Zhang, Liang Yu, Song Chen

**Affiliations:** 1 Department of Central Laboratory, The Affiliated Huaian No. 1 People's Hospital, Nanjing Medical University, Huai'an, Jiangsu, 223300, China.; 2 Department of Hematology, The Affiliated Huaian No.1 People's Hospital,Nanjing Medical University, Huai'an, Jiangsu, 223300, China.; 3 Institute of Medicinal Biotechnology, Jiangsu College of Nursing, Huai'an, Jiangsu, 223300, China.; 4 The Chinese Academy of Sciences (CAS), Key Laboratory of Innate Immunity & Chronic Disease, CAS Center for Excellence in Cell & Molecular Biology, School of Life Sciences, University of Science & Technology of China, Hefei 230026, China.; 5 Department of Oncology, Nanjing First Hospital, Nanjing Medical University, Nanjing, 210006, China.; 6 Translational Research Institute of Henan Provincial People's Hospital and People's Hospital of Zhengzhou University, and Molecular Pathology Center, Academy of Medical Sciences, Zhengzhou University, Zhengzhou, Henan 450053, China.; 7 School of Environmental and Life Sciences, The University of Newcastle, NSW, 2258, Australia.; 8 School of Biomedical Sciences and Pharmacy, The University of Newcastle, NSW, 2308, Australia.

**Keywords:** KDM6A/UTX, YY1, *NTRK1*/TRKA, CML, imatinib resistance

## Abstract

**Rationale:** Despite landmark therapy of chronic myelogenous leukemia (CML) with tyrosine kinase inhibitors (TKIs), drug resistance remains problematic. Cancer pathogenesis involves epigenetic dysregulation and in particular, histone lysine demethylases (KDMs) have been implicated in TKI resistance. We sought to identify KDMs with altered expression in CML and define their contribution to imatinib resistance.

**Methods:** Bioinformatics screening compared KDM expression in CML versus normal bone marrow with shRNA knockdown and flow cytometry used to measure effects on imatinib-induced apoptosis in K562 cells. Transcriptomic analyses were performed against KDM6A CRISPR knockout/shRNA knockdown K562 cells along with gene rescue experiments using wildtype and mutant demethylase-dead KDM6A constructs. Co-immunoprecipitation, luciferase reporter and ChIP were employed to elucidate mechanisms of KDM6A-dependent resistance.

**Results:** Amongst five KDMs upregulated in CML, only KDM6A depletion sensitized CML cells to imatinib-induced apoptosis. Re-introduction of demethylase-dead KDM6A as well as wild-type KDM6A restored imatinib resistance. RNA-seq identified NTRK1 gene downregulation after depletion of KDM6A. Moreover, NTRK1 expression positively correlated with KDM6A in a subset of clinical CML samples and KDM6A knockdown in fresh CML isolates decreased NTRK1 encoded protein (TRKA) expression. Mechanistically, KDM6A was recruited to the NTRK1 promoter by the transcription factor YY1 with subsequent TRKA upregulation activating down-stream survival pathways to invoke imatinib resistance.

**Conclusion:** Contrary to its reported role as a tumor suppressor and independent of its demethylase function, KDM6A promotes imatinib-resistance in CML cells. The identification of the KDM6A/YY1/TRKA axis as a novel imatinib-resistance mechanism represents an unexplored avenue to overcome TKI resistance in CML.

## Introduction

Chronic myelogenous leukemia (CML) is characterized by the Philadelphia (Ph) chromosome that results in the expression of the constitutively active tyrosine kinase BCR-ABL 1. Targeting BCR-ABL using tyrosine kinase inhibitors (TKIs) has become the standard of care for CML patients 2. Treatment with imatinib, the first of this class of drugs, can achieve a complete hematologic and cytogenetic response, but a multitude of patients eventually develop resistance or intolerance 3. A major mechanism responsible for the resistance of CML to imatinib is point mutations in the *BCR-ABL* kinase domain 4. Although second-generation TKIs including dasatinib and nilotinib can overcome the resistance caused by many imatinib-resistant mutants, they remain ineffective against the T315I “gatekeeper” mutation 5. The third-generation TKI ponatinib can potently overcome the resistance caused by T315I and other *BCR-ABL* mutations, but its application is frequently complicated by unacceptable cardiovascular toxicity associated with its broad-spectrum inhibition profile 6. It seems therefore that an alternative strategy to overcome resistance of CML to TKIs is to target downstream molecular modules essential for CML cell survival.

There is increasing evidence that indicates that epigenetic dysregulation is involved in the pathogenesis of cancer 7, 8. This has led to the development of drugs targeting DNA methyltransferases and histone methyltransferases 9. Another class of epigenetic modifiers that are emerging as molecular targets for cancer treatment are histone lysine demethylases (KDMs), which constitute two broad family divisions: the lysine-specific demethylases represented by KDM1A and KDM1B that act on mono- and dimethylated lysines at lysine 4 or lysine 9 of H3 and the Jumanji (JmjC) domain-containing KDMs that contains five subfamilies (KDM2-7) that catalyse the demethylation of mono-, di- and trimethylated lysines in both histones and non-histone substrates 10. Of the latter, KDM6A (also known as UTX) has been shown to be important for embryogenesis as female mice homozygous for catalytically inactive KDM6A have severe developmental defects 11. Mechanistically, KDM6A functions as a component of the MLL3/4-COMPASS (complex of proteins associated with Set1)-like complex to co-activate gene transcription likely through removing repressive lysine 27 histone 3 methylation marks 12, 13. Nevertheless, KDM6A may also act independently of its demethylase activity 14. KDM6A has also been broadly implicated as a tumor suppressor gene where its mutational loss commonly occurs in cancer cell lines of diverse tissue origins 15. KDM6A mutations have been confirmed in corresponding patient samples 16, 17, for example, over 30% of bladder cancers contain KDM6A mutations and these mutations also occur to a lesser extent in hematological malignancies including acute lymphoblastic leukemia (subtypes of T-cell and B-cell ALL) along with chronic myelomonocytic leukemia (CMML) 18, 19. Conversely, some KDMs including KDM6A have been shown to be upregulated in human primary acute myelogenous leukemia (AML) cells and inhibiting histone demethylase activity in these cells reduces their survival 20. In this report, we have explored the potential roles of KDMs in resistance of CML to imatinib. We show here that KDM6A is commonly upregulated in CML cells and its expression is important for CML cell survival upon treatment with imatinib. Notably, KDM6A functions independently of its demethylase activity to promote YY1-mediated transcriptional upregulation of TRKA. Moreover, we demonstrate that KDM6A-mediated activation of TRKA is required for protection of CML cells against imatinib afforded by the neurotrophin nerve growth factor (NGF). These results suggest that targeting KDM6A represents a useful strategy for overcoming resistance of CML to TKIs.

## Results

### KDM6A is upregulated and confers resistance to imatinib in CML cells

**Figure 1:**
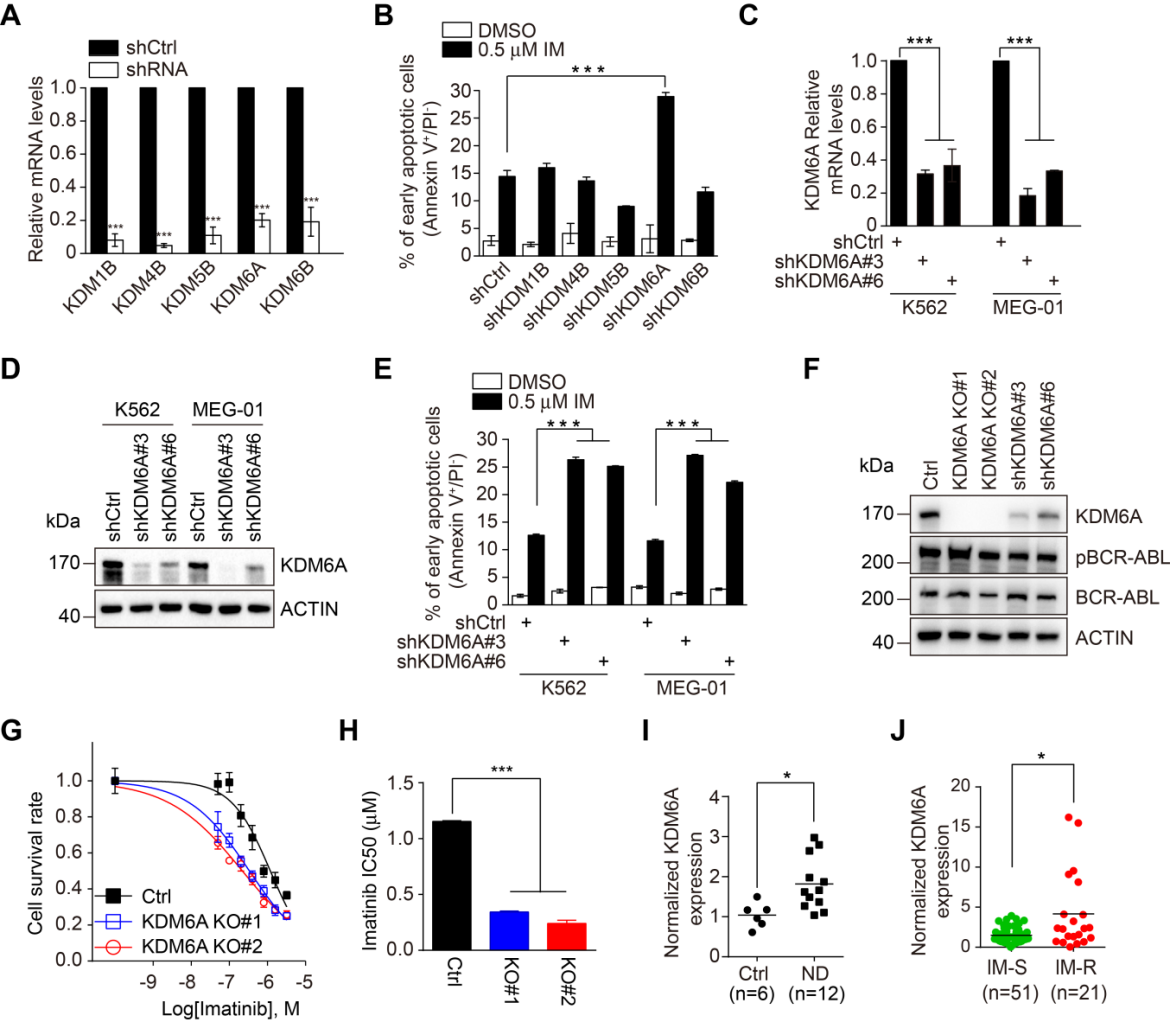
**KDM6A is upregulated in CML and depletion of KDM6A sensitizes CML cells to imatinib-induced apoptosis. (A)** Knockdown efficiency of shRNA-bearing lentiviruses against individual KDMs in K562 cells compared to a scrambled shRNA control. Cells were transduced for 72 h and relative expression determined by qPCR. shKDM6A: shKDM6A#3. Mean ± s.d. are given for three independent experiments. Unpaired, two-tailed Student's t-test; ***p < 0.001. **(B)** The rates of apoptosis in shCtrl versus individual shKDM K562 cells after 24 h treatment with DMSO vehicle control or 0.5 μM imatinib. The percentage of early apoptotic cells was determined by FACS using annexin V/PI double staining. shKDM6A: shKDM6A#3. Mean ± s.d. are given for three independent experiments. Unpaired, two-tailed Student's t-test; ***p < 0.001. **(C, D)** Knockdown efficiency of two independent shRNAs against KDM6A in K562 and MEG-01 cells stably transduced with lentivirus was determined by qPCR **(C)** and Western blotting** (D)**. Mean ± s.d. are given for three independent experiments. One-way ANOVA; ***p < 0.001. **(E)** The rates of apoptosis in shCtrl versus shKDM6A K562 cells and MEG-01 cells were determined by FACS after treatment with 0.5 μM imatinib for 24 h. Mean ± s.d. are given for three independent experiments. One-way ANOVA; ***p < 0.001. **(F)** Western blotting analysis against KDM6A, pBCR/ABL, BCR/ABL in K562 control, KDM6A KO#1 and KO#2 cells, shKDM6A#3 and shKDM6A#6. **(G, H)** Imatinib dose-response effects on cell viability were measured in control versus KDM6A knockout cells after 48 h using the Cell Counting Kit-8 (CCK-8) assay. The data points were fitted by nonlinear regression analysis using GraphPad Prism 6. **(H)**. Comparison of IC50 values for imatinib in K562 control versus KDM6A knockout cells. Mean ± s.d. are given for three independent experiments. One-way ANOVA; ***p < 0.001. **(I, J)** Comparisons of KDM6A mRNA levels in bone marrow between 6 control patients (idiopathic thrombocytopenic purpura) and 12 newly diagnosed CML patients (ND) (H) and between PBMC from CML sensitive (IM-S) and resistant (IM-R) CML patients **(I)** determined by qPCR. Horizontal bar shows mean. Unpaired, two-tailed Student's t-test **(I)**; Mann-Whitney test **(J)**; *p <0.05.

Through interrogating datasets acquired from Oncomine, we derived a list of KDMs that were upregulated in CML in comparison with either normal bone marrow or peripheral blood mononuclear cells (PBMCs), including KDM1B, KDM4B, KDM5B, KDM6A and KDM6B (Figure S1A-B). Strikingly, although shRNA knockdown of the individual KDMs did not impinge on the viability of K562 CML cells that harbor wild-type BCR-ABL (Figure 1A), knockdown of KDM6A but not the other KDMs rendered K562 cells more sensitive to apoptosis induced by imatinib (Figure 1B and Figure S1C). This effect of KDM6A knockdown was confirmed using two independent shRNAs (Figure 1C-E). Moreover, K562 cells with KDM6A knocked out using the lentiCRISPR v2/Cas9 system (Figure S1D) appeared markedly more sensitive to imatinib than control counterparts (Figure 1F-G). This significantly reduced the IC50 for imatinib from 1.15 to 0.24 μM (Figure 1H) indicating that KDM6A plays a role in protecting CML cells against imatinib. Depletion of KDM6A by either knockout or shRNAs caused no changes in the expression of BCR-ABL or its phosphorylation at Tyr177 (Figure 1F), indicating that protection of CML cells against imatinib by KDM6A is not due to alterations in the expression or activity of BCR-ABL. The role of KDM6A in protection of CML cells against imatinib is not cell line dependent as knockdown of KDM6A also sensitized MEG-01 CML cells that carry wild-type BCR-ABL to imatinib-induced apoptosis (Figure 1E and Figure S2A). Notably in the absence of imatinib treatment, depletion of KDM6A in both K562 and MEG-01 cells had no appreciable effect on cell growth (Figure S2B-C).

Interrogation of bone marrow samples from newly diagnosed CML patients against control patients with idiopathic thrombocytopenic purpura showed an increase in KDM6A mRNA expression associated with CML (Figure 1I and Table S1). Moreover, a group of bone marrow samples from patients who were refractory to imatinib treatment displayed even higher levels of KDM6A mRNA than those who responded to the treatment (Figure 1J and Table S1). The general increase in KDM6A in CML along with the high levels observed in imatinib refractory CML cases proposes that the KDM6A-dependent mechanism of imatinib resistance is also active in patients.

### KDM6A protects CML cells against imatinib independently of its demethylase activity

**Figure 2:**
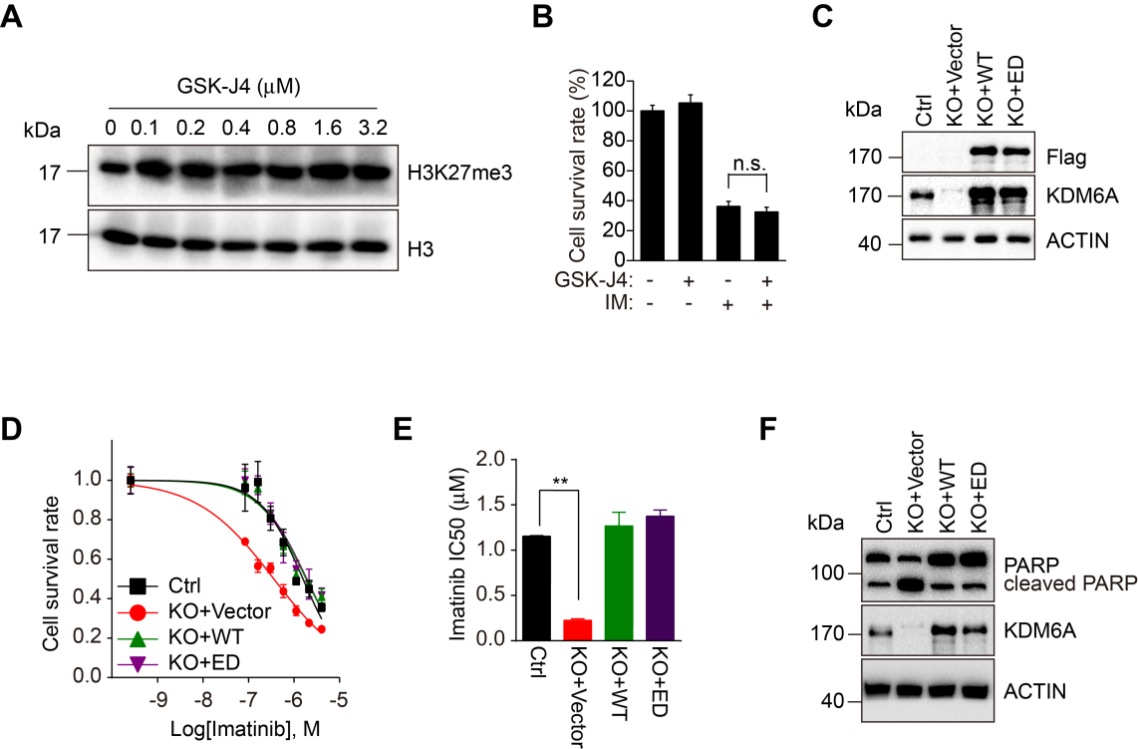
** KDM6A enhances cell viability upon imatinib treatment independently of its enzymatic activity. (A)** K562 cells were treated with different concentration of GSK-J4 inhibitor as indicated or DMSO vehicle control for 24 h. Histones were acid extracted as described in Methods before Western blotting analysis against H3K27me3. **(B)** Cell viability of K562 cells exposed to 0.5 μM imatinib and/or 100 nM GSK-J4 inhibitor determined using the CCK-8 assay. Mean ± s.d. are given for three independent experiments. Unpaired, two-tailed Student's t-test; n.s., not significant. **(C)** Reconstitution of K562 KDM6A KO#2 cells with Flag-wild-type KDM6A(WT) or the enzymatic-dead mutant (ED) was confirmed by Western blotting against Flag and KDM6A. **(D, E)** Imatinib dose-response curves for control (parental) K562 versus KDM6A KO#2 cells and KDM6A-reconstituted cells from **(C)** determined after 48 hours using the CCK-8 assay. **(E)** Comparison of IC50 values for imatinib in K562 control versus KDM6A KO#2 cells reconstituted with vector (KO+vector), KDM6A (KO+WT) or KDM6A-ED (KO+ED). Mean ± s.d. are given for three independent experiments. Unpaired, two-tailed Student's t-test; **p < 0.01. **(F)** Western blotting analysis showing increased cleaved PARP in KDM6A KO cells treated with 1μM imatinib for 48 h but not in KDMA6A or KDM6A-ED reconstituted cells.

Since the biological roles of KDMs are predominantly but not exclusively dependent on their demethylase activities, we investigated whether the demethylase activity of KDM6A is required for its protection of CML cells against imatinib. We employed GSK-J4, an inhibitor affecting the demethylase activity of KDM6A and related demethylases 21, 22. Dose titration of GSK-J4 against K562 cells identified 100 nM could optimally increase global H3K27me3 levels without adversely affecting cell growth (Figure 2A and Figure S2D). However, GSK-J4 treatment did not affect the sensitivity of K562 cells to imatinib (Figure 2B), implying that the demethylase activity of KDM6A is dispensable for protection of CML cells against imatinib. To verify this notion, we introduced an enzymatically-dead KDM6A mutant (KDM6A-ED) carrying H1146A and E1148A mutations into K562 cells with endogenous KDM6A knocked out (Figure 2C) 14. While re-expression of wild-type KDM6A restored resistance to imatinib, the expression of the KDM6A-ED similarly rescued the imatinib-sensitive phenotype (Figure 2D-F). Thus, KDM6A protects CML cells from imatinib independently of its demethylase activity.

### KDM6A protects CML cells against imatinib through TRKA

**Figure 3:**
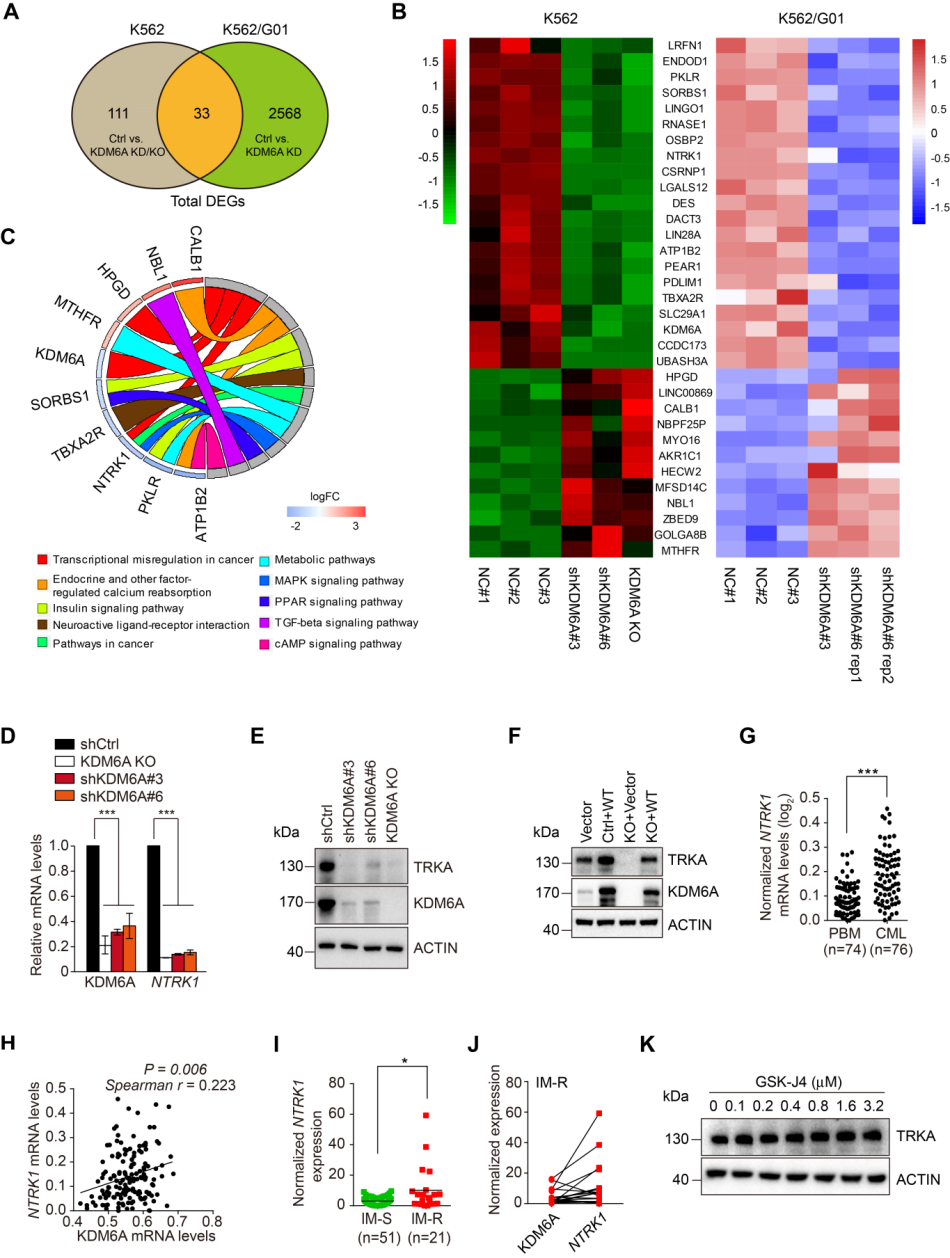
** KDM6A regulates TRKA expression in CML cells. (A)** Venn diagram illustrated overlap in differentially expressed genes in KDM6A depletion in K562 cells and K562/G01 cells. The DEGs were identified using ballgown package in R with a cut-off criteria of adjusted *p*<0.05 and |log2fold change| > 0.6. **(B)** Heatmap of the gene list containing shared altered genes in (**A**). **(C)** KEGG pathway analysis of the KDM6A regulated genes in **(B)** using GOplot package in R. **(D, E)** NTRK1/TRKA down-regulation accompanies KDM6A inhibition. qPCR analyses conducted against KDM6A and NTRK1 mRNA expression in shCtrl, KDM6A KO#2, shKDM6A#3 and shKDM6A#6 K562 cells **(D)**. Western blot analysis of TRKA and KDM6A in cells from **(E)**. Mean ± s.d. are given for three independent experiments. One-way ANOVA; ***p < 0.001. **(F)** Western blot showing re-expression of KDM6A in K562 KDM6A KO#2 cells rescues TRKA expression. **(G-J)** *NTRK1* and KDM6A expression in clinical CML samples. NTRK1 RNA expression levels are compared in PBMC from heathy donors and CML patients from the Oncomine dataset** (G)** Comparative expression between NTRK1 and KDM6A in CML samples from** (G)** analysed by Spearman correlation **(H)**. Comparison of *NTRK1* mRNA levels between imatinib-sensitive (IM-S) and imatinib-resistant CML patients (IM-R) without T315I mutation **(I)** and pairwise comparisons between KDM6A and NTRK1 expression in the IM-R patients **(J)**. Horizontal bar shows mean. Mann-Whitney test **(G, I)**; *p <0.05; ***p < 0.001. **(K)** Western blot showing inhibition of KDM6A demethylase activity by 24 h treatment with different concentration of GSK-J4 has no effect on the TRKA expression in K562 cells.

To identify genes and signaling pathways associated with the KDM6A-dependent protection of CML cells against imatinib, we carried out comparative transcriptomic analyses of K562 cells and its imatinib-resistant subline K562/G01 both with and without KDM6A depletion. These analyses identified 144 differentially regulated genes (DEGs) in K562 cells before and after KDM6A depletion with 2601 DEGs in K562/G01 cells (Figure S3A-B, respectively). Application of the 33 genes in common to hierarchical clustering analysis of gene expression profiles showed ten genes were specifically enriched in KEGG pathways with KDM6A associated with 'transcriptional misregulation in cancer'(Figure 3A-B). Strikingly, downregulation of *NTRK1* encoding tropomyosin receptor kinase A (TRKA), a high affinity receptor for nerve growth factor (NGF), was most prominently associated with three KEGG pathways, namely 'transcriptional misregulation in cancer', 'Pathways in cancer' and the 'MAPK signaling pathway' (Figure 3C). Alternative analysis of the DEGs using GSEA obtained a top ranked list of six enriched pathways including two annotated pathways that include NTRK1, namely 'Neuroactive_Ligand_Receptor_Interaction' and “Thyroid Cancer' (Figure S3C). The association of TRKA with these pathways proposed its potential involvement in resistance to imatinib.

**Figure 4:**
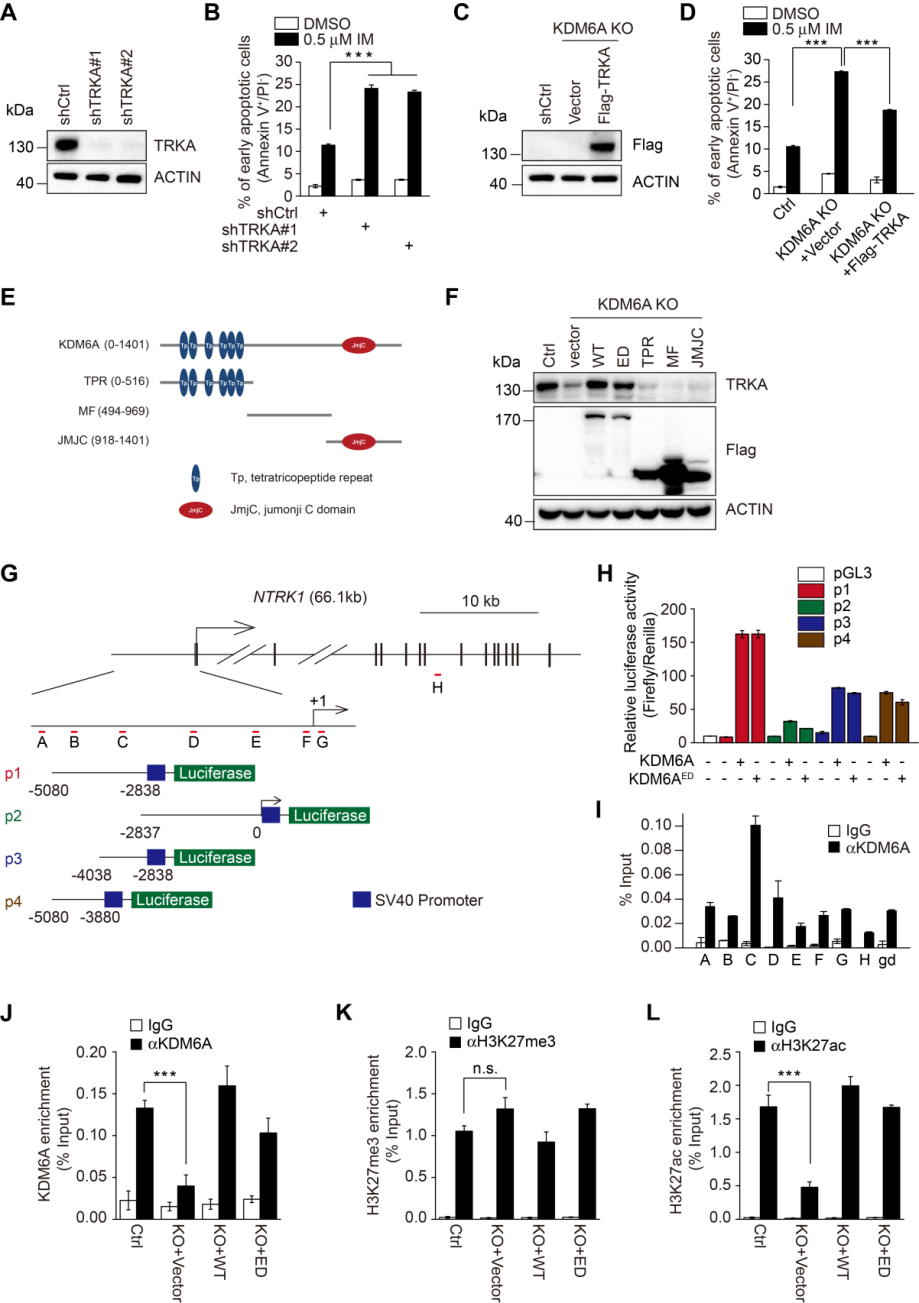
** KDM6A promotes imatinib-resistance through transcriptional activation of NTRK1. (A)** Knockdown efficiency of two independent shRNAs targeting TRKA in K562 cells as determined by Western blot. **(B)** The rates of early apoptosis in shCtrl versus shTRKA K562 cells were determined by FACS after treatment with 0.5 μM imatinib for 24 h. Mean ± s.d. are given for three independent experiments. One-way ANOVA; ***p < 0.001. **(C, D)** Western blot showing stable expression of Flag-TRKA but not Flag vector control restores TRKA expression in K562 KDM6A KO#2 cells **(C)** and significantly decreases their imatinib sensitivity **(D)** as shown by decreases in apoptotic cells after treatment with 0.5 μM imatinib for 24 h. Mean ± s.d. are given for three independent experiments. One-way ANOVA; ***p < 0.001. **(E, F)** Schematic diagram of KDM6A protein (top) and the design of domain truncation mutants (bottom; **E**). Flag-tagged versions wild-type KDM6A (WT), an enzyme-dead mutant of KDM6A (ED) or the KDM6A truncation mutants along with a flag-vector control were used as lentiviral constructs to stably infected KDM6A KO#2 cells **(F)**. The expression of transduced proteins was revealed by Western blotting against Flag along with changes in TRKA expression. **(G)** Promoter region and intron/exon organization of *NTRK1.* Schematic shows the four overlapping fragments (p1, p2, p3 and p4) used to construct pGL3-promoter based luciferase reporters along with the position of the amplicon targets (A-H) used in ChIP-qPCR assays. **(H)** Luciferase reporter assays conducted in U2OS cells transfected with the indicated pGL3-*NTRK1* reporter vectors from **(G)** in combination with KDM6A or KDM6A-ED. **(I)** ChIP-qPCR analyses against the *NTRK1* gene using the amplicon targets shown in** (H)** along with a control gene desert sequence (*gd*) Chr2(q36.3). Assays were performed in K562 cells using either control IgG or anti-KDM6A antibody. Mean ± s.d. are given for three independent experiments. Unpaired, two-tailed Student's t-test; ***p < 0.001. **(J-L)** ChIP-qPCR assays were conducted as per **(I)** against KDM6A** (J)**, H3K27me3 **(K)**, or H3K27ac **(L)** against amplicon targets C, comparing parental K562 cells (Ctrl) against KDM6A KO#2 cells reconstituted with vector (KO+vector), KDM6A (KO+WT) or KDM6A-ED (KO+ED). **(A-D, F, H-L)** represent three independent experiments. Mean ± s.d. are given for three independent experiments. Unpaired, two-tailed Student's t-test; ***p < 0.001; n.s., not significant.

We confirmed that NTRK1 mRNA and protein (TRKA) were downregulated after KDM6A knockout/knockdown in K562 cells using qPCR (Figure 3D) and Western blotting (Figure 3E). Similarly, knockdown of KDM6A resulted in downregulation of TRKA at both the mRNA and protein levels in MEG-01 cells (Figure S3D-E). Consistently, re-expression of wildtype KDM6A in K562 cells with KDM6A knocked out rescues TRKA expression and overexpression of KDM6A caused, albeit moderately, upregulation of TRKA in K562 cells (Figure 3F). Together, these results indicate that KDM6A drives the expression of TRKA in CML cells. Substantiating this, the expression levels of TRKA were upregulated and positively correlated with the levels of KDM6A in clinical CML samples (Figure 3G-J). As anticipated, treating K562 cells with GSK-J4 to inhibit the demethylase activity of KDM6A did not impinge on TRKA expression (Figure 3K). Moreover, ectopic expression of the KDM6A-ED mutant, similar to re-expression of wild-type KDM6A, restored TRKA expression (Figure 4F).

In agreement with previous reports that TRKA signaling protects CML cells from imatinib 23. knockdown of TRKA by shRNAs enhanced sensitivity of K562 cells to imatinib-induced apoptosis (Figure 4A-B and Figure S4A). To test whether TRKA is required for protection of CML cells by KDM6A, we introduced exogenous TRKA into K562 cells with KDM6A knocked out (Figure 4C). Indeed, ectopic expression of TRKA partially rescued the imatinib-senstitive phenotype caused by knockout of KDM6A (Figure 4D and Figure S4B). Therefore, TRKA is the functional effector of KDM6A in protection of CML cells against imatinib.

### KDM6A functions as a transcriptional activator of *NTRK1*

Having established that KDM6A promotes the expression of TRKA independently of its demethylase activity, we tested whether other functional domains of KDM6A are necessary for its effect on TRKA expression. Introduction of the KDM6A truncation mutant with the tetratricopeptide repeat (TPR), Jumanji (JMJC) domains or MF (middle fragment) into K562 cells with KDM6A knocked out (Figure 4E and Figure S5A-B) failed to restore the expression of TRKA (Figure 4F), suggesting that intact KDM6A is necessary for upregulation of TRKA.

To examine how KDM6A promotes TRKA expression, the ~5 kb upstream region of the NTRK1 proximal promoter was divided into two over-lapping segments designated p1 and p2 and these were cloned into luciferase reporter plasmids (Figure 4G). Co-introduction of p1 or p2 construct with KDM6A demonstrated that KDM6A strongly enhanced the transcriptional activity of the p1 reporter which encompasses the -5080/-2838 region (Figure 4H), whereas it had a minimal effect on the p2 reporter that spanned -2837 bp upstream of the transcription start site (TSS). Instructively, luciferase reporter results obtained with the KDM6A-ED mutant phenocopied those of wildtype KDM6A (Figure 4H). Further subdividing p1 into two further fragments demonstrated that both constructs (p3 and p4) were responsive to *NTRK1* expression, albeit with approximately half the magnitude of response compared to p1 (Figure 4H). These data suggest that a KDM6A functional site exists within the -5080/-2838 region of *NTRK1*. In support, chromatin immunoprecipitation (ChIP) assays conducted against target sequences across the NTRK1 promoter showed that KDM6A was most strongly enriched with the C-primers which amplifies sequences within the -4038 to -2837 region common to the p1, p3 and p4 fragments (Figure 4I). To further confirm the ability of the demethylase dead KDM6A mutant to bind to the NTRK1 promoter and influence transcription, we repeated the ChIP-qPCR assays in K562-KO cells reconstituted with either wildtype or KDM6A-ED. Ectopically expressed KDM6A and KDM6A-ED both significantly bound to -4038 to -2837 region but not a control region (Figure 4J and Figure S6A-B). Moreover, there were no significant changes in histone tri-methylation observed in ChIP assays against H3K27me3 (Figure 4K and Figure S6C-D) and nor were there global changes in H3K27me3 after KDM6A knockout (Figure S6G). Similarly, the global levels of of histone acetylation were also unchanged (Figure S6G). Interestingly the H3K27ac ChIP-qPCR levels associated with the NTRK1 promoter were reduced following KDM6A knockout but could be restored by re-expression of either KDM6A wildtype or ED mutant proteins (Figure 4L and Figure S6E-F). Based on a previous report showing the histone acetylase CBP (p300) associated with KDM6A to affect changes in H3K27ac levels 24, we further checked if CBP associated with KDM6A in K562 cells. However, no associated CBP was observed in KDM6A immunoprecipitates although as expected, enrichment of RNA Pol II was observed (Figure S6H). Collectively, these results establish that KDM6A acts as a transcriptional activator of *NTRK1* independently of its demethylase activity.

### KDM6A is necessary for YY1-mediated transcription of *NTRK1*

**Figure 5:**
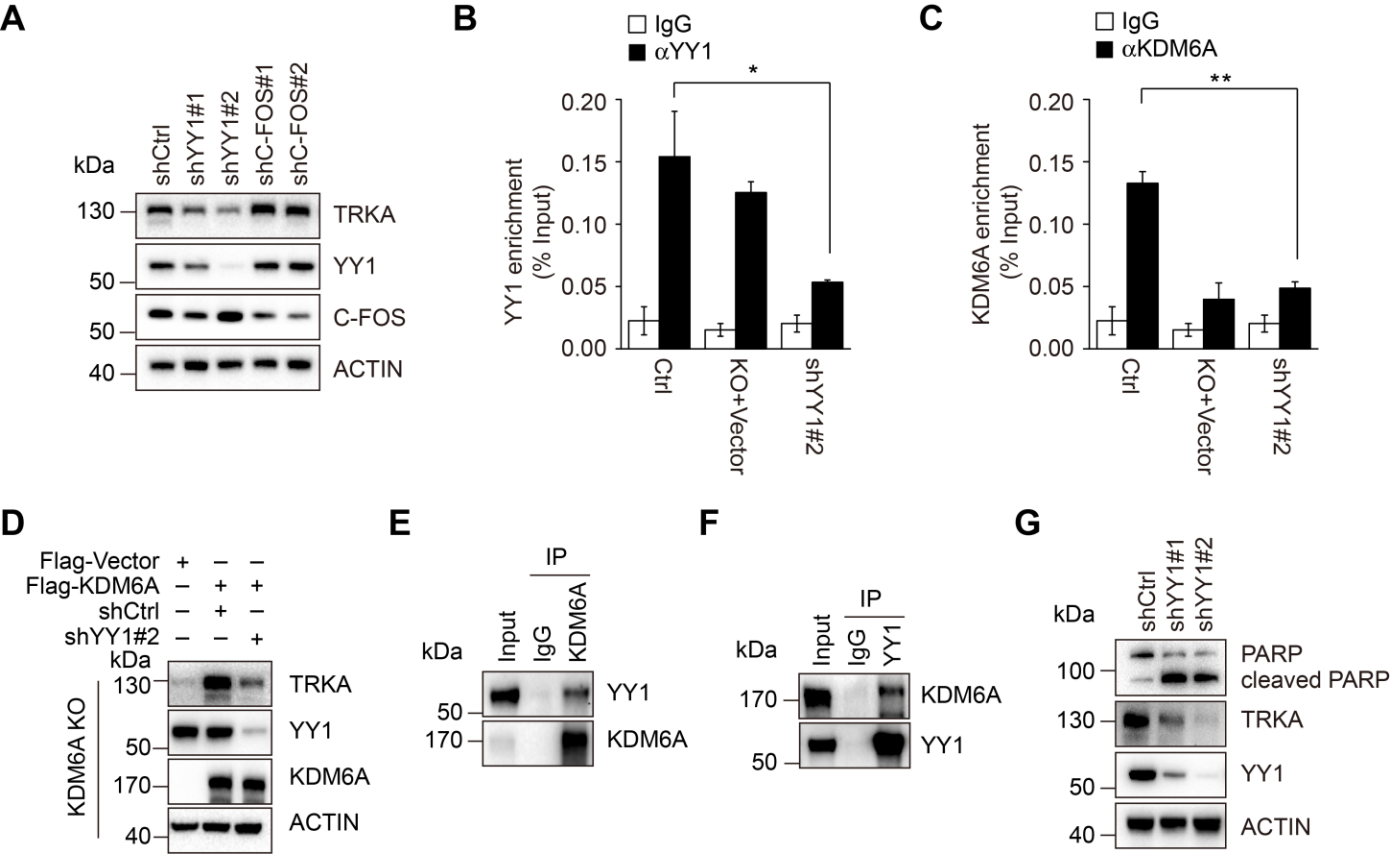
** YY1 is necessary for KMD6A-mediated *NTRK1* transcription. (A)** Western blot analysis showing knockdown of YY1 but not c-Fos by lentiviral-mediated shRNA transduction decreased TRKA protein expression in K562 cells. **(B, C)** ChIP-qPCR assays were conducted using antibodies against YY1 **(B)** or KDM6A **(C)** in control K562 versus KDM6A-KO cells or cells expressing shRNA against YY1. Target amplicons targeting C region are illustrated in Fig. 4G. KDM6A-KO failed to significantly reduce recovery of YY1 ChIP signals at the NTRK1 enhancer region **(B)** whereas conversely YY1 knockdown reduced KDM6A ChIP target recovery **(C)**. Mean ± s.d. are given for three independent experiments. Unpaired, two-tailed Student's t-test; *p <0.05. **(D)** Western blot analysis showing re-constitution of TRKA expression in KDM6A KO K562 cells requires YY1. **(E, F)** Immunoprecipitation analyses conducted against KDM6A **(E)** or YY1** (F)** in K562 cells demonstrates reciprocal co-immunoprecipitation between KDM6A and YY1. IgG was used as a control. **(G)** Western blot analysis showing increased PARP cleaved in K562 cells after treatment with 1 μM imatinib IM for 48 h when YY1 is depleted.

To clarify the mechanism through which KDM6A promotes transcriptional activation of *NTRK1*, bioinformatics analyses conducted on the *NTRK1* promoter determined that the -5080/-2838 fragment was enriched in binding sites for the transcription factors YY1 and C-FOS. Instructively, knockdown of YY1 but not c-FOS led to downregulation of TRKA expression (Figure 5A), suggesting that YY1 plays an important role in transcriptional activation of *NTRK1*. ChIP assays revealed that YY1 indeed bound to the -4038/-2838 fragment (Figure 5B and Figure S6I-J). We next examined the relationship between YY1, KDM6A and the transcriptional regulation of *NTRK1*. Knockdown of YY1 resulted in a dramatic decrease in KDM6A binding to the NTRK1 promoter (Figure 5C and Figure S6K). Moreover, re-expression of wild-type KDM6A could not restore the expression of TRKA in K562 cells with deletion of KDM6A and YY1 knockdown (Figure 5D), indicating that KDM6A-dependent transcriptional upregulation of TRKA requires YY1. Intriguingly, co-immunoprecipitation assays demonstrated binding between KDM6A and YY1 (Figure 5E-F), suggesting they are components of a complex necessary for transcriptional regulation of *NTRK1*.

We also tested the importance of YY1 in KDM6A-mediated protection of CML cells against imatinib. Knockdown of YY1 sensitized CML cells to imatinib-induced apoptosis akin to the effect of KDM6A knockdown (Figure 5G). Thus, YY1 is necessary for protection of CML cells against imatinib by KDM6A.

### KDM6A-mediated activation of *NTRK1* is required for NGF protection of CML cells against imatinib

**Figure 6:**
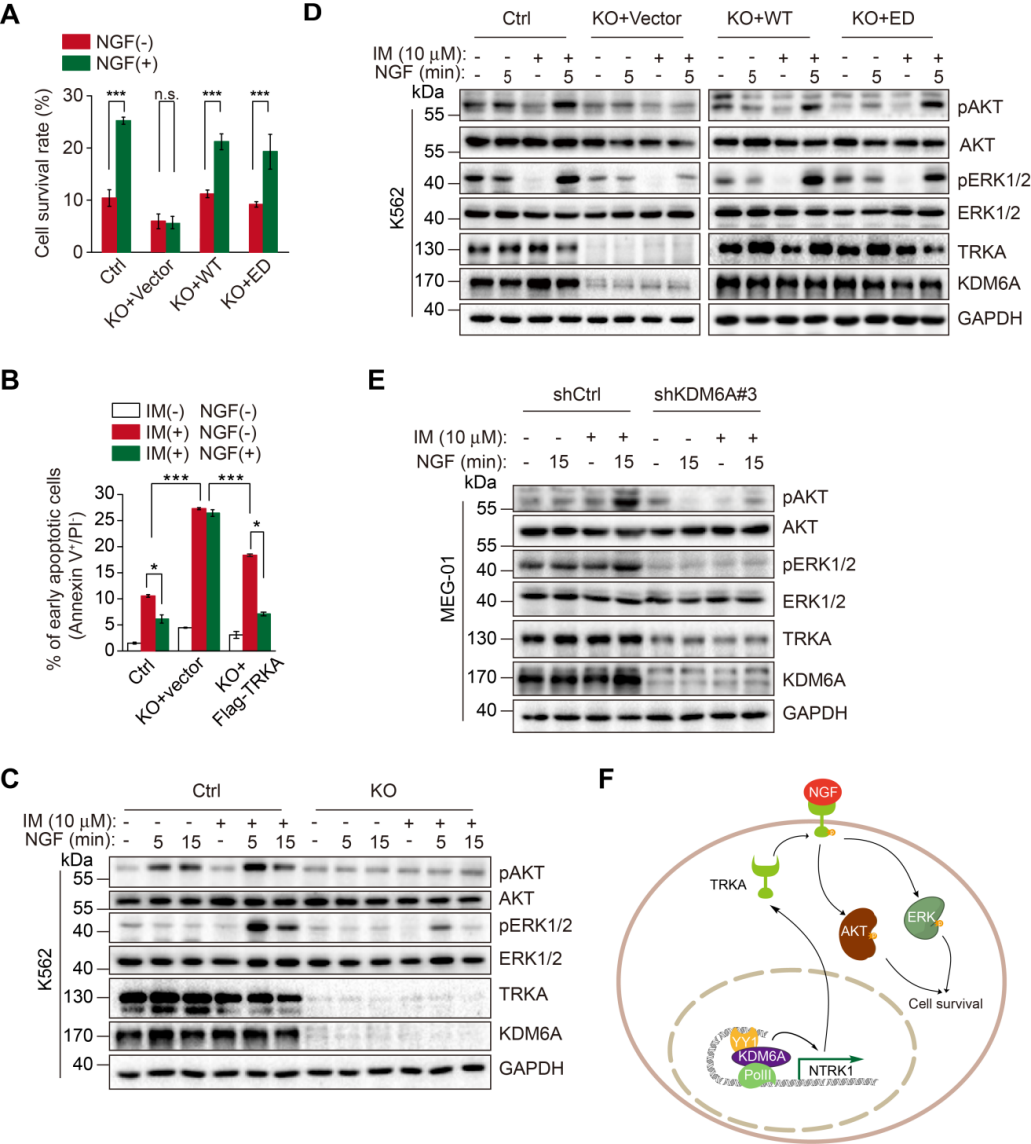
** NGF protects CML cells against imatinib through KDM6A-mediated activation of TRKA. (A)** Cell survival comparisons between K562 (Ctrl), KDM6A KO and KDM6A KO cells rescued by either WT or ED KDM6A constructs after 3 days of growth in 10% FBS containing medium supplemented with or without NGF (100 ng/mL) in the presence of 0.5 μM imatinib. Live cells were counted by Trypan blue exclusion and results calculated as a percentage of growth seen with untreated controls. Mean ± s.d. are given for three independent experiments. Unpaired, two-tailed Student's t-test; ***p < 0.001; n.s., not significant. **(B)** Ectopic Flag-TRKA expression in K562 KDM6A KO cells increased NGF-induced cell survival after 24 h treatment with 0.5 μM imatinib. Apoptosis was determined by FACS. Mean ± s.d. are given for three independent experiments. Unpaired, two-tailed Student's t-test; *p < 0.05. One-way ANOVA; ***p < 0.001. **(C-E)** Western blotting analyses comparing AKT and ERK signaling responses elicted by 100 ng/mL NGF and/or 1 h pretreatment with 10 μM imatinib. K562 control versus KDM6A KO K562 cells** (C)**, KDM6A KO K562 cells after reconstitution with KDM6A WT or KDM6A-ED **(D)** and parental MEG-01 CML versus KDM6A knock down cells **(E)**. **(F)** A schematic showing KDM6A mediated signaling responses directing imatinib resistance in CML.

We examined the role of KDM6A-mediated activation of NTRK1 in resistance of CML cells to imatinib caused by NGF 23. Knockout of KDM6A diminished the protection of K562 cells against imatinib afforded by treatment with NGF (Figure 6A). However, re-expression of wild-type KDM6A or introduction of the KDM6A-ED mutant rescued the protective effect (Figure 6A), recapitulating the effect of ectopic expression of TRKA in KDM6A KO cells (Figure 6B and Figure S4B). These results indicate that KDM6A-driven expression of TRKA is needed for resistance of CML cells to imatinib triggered by NGF. Mechanistically, KDM6A knockout abolished NGF-induced activation (phosphorylation) of Akt and ERK1/2 (Figure 6C), which are known to be involved in NGF-mediated protection of CML cells against imatinib 23, whereas re-expression of wild-type KDM6A or introduction of the KDM6A-ED mutant re-enabled NGF to trigger activation of Akt and ERK1/2 in K562 cells with KDM6A knocked out (Figure 6D). Moreover, the effects of KDM6A on NGF-triggered activation of Akt and ERK1/2 were also observed in MEG-01 cells with KDM6A knocked down by shRNA (Figure 6E). Similar to these findings in K562 and MEG-01 cells, knockdown of KDM6A in KU812 CML cells as well as the imatinib-resistant K562/G01 subline was able to abrogate the protective effects of NGF after treatment with imatinib (Figure S7A-C and Figure S7D-F, respectively).

Lastly, to confirm that the KDM6A-TRKA mechanism revealed in the cell line experiments was relevant to CML *in vivo*, we undertook knockdown of KDM6A in fresh bone marrow isolates from three newly diagnosed CML patients (Table S1). Importantly, depletion of KDM6A decreased TRKA expression in all three samples (Figure S8A), providing proof of concept that the regulatory axis exists in *bona fide* CML cells. NGF did not rescue the cells from increased apoptotic cell death when KDM6A was knocked down (Figure S8B-C), indicating KDM6A intrinsically confers resistance to imatinib through TRKA in CML patients.

## Discussion

Despite the recent advances in the development of novel targeted therapies, drug resistance along with intolerance remains a major obstacle for curative treatment of CML using TKIs. The above results demonstrate that the increased expression of KDM6A plays a role in resistance of CML cells to the TKI imatinib. Noticeably, this effect of KDM6A is not due to its demethylase activity. Instead, KDM6A acts as a partner of the transcription factor YY1 to promote transcriptional activation of TRKA in CML cells (Figure 6F). Regardless, these results point to the potential of targeting KDM6A in overcoming resistance of CML to TKIs, in particular, when the resistance is conferred by NGF signaling.

Since inactivating mutations in the* KDM6A* gene are found in a number of human cancers and its inactivation invokes oncogenic phenotypes in various experimental systems, it is thought that KDM6A primarily functions as a tumor suppressor 15, 18, 25, 26. Moreover, KDM6A is highly expressed in hematopoietic stem and progenitor lineages 27, suggestive of its importance in the hematopoietic compartment. Indeed, mice deficient in *Kdm6a* exhibit disturbances in normal hematopoiesis and develop acute myelogenous leukemia, albeit with a long latency 28. Furthermore, loss of KDM6A has recently been reported to confer drug resistance in human AML cells 29. On the other hand, KDM6A also displays an oncogenic role in varying settings 30, 31. For example, it not only acts as tumor suppressor in certain T-ALL cases 32, but KDM6A overexpression in TAL1-expressing T-ALL increasesd their growth 33. Similarly, KDM6B which is the most closely homologous KDM to KDM6A 34 is known to be essential for the initiation and maintenance of T-ALL by modulating H3K27 methylation 32. Furthermore, high KDM6A expression was associated with worse survival of breast cancer patients and overexpression of KDM6A promoted breast cancer cell growth *in vitro* 35, 36. It seems therefore that KDM6A impinges on cancer pathogenesis in a highly tissue type- and context-dependent fashion.

Here we found that five KDMs were upregulated in CML cells derived from patients compared with normal bone marrow cells including KDM6A and KDM6B. However, only knockdown of KDM6A rendered cultured CML cells more sensitive to killing by imatinib. Moreover, this mechanism was shown to be relevant to imatinib-selected CML cells expressing elevated levels of BCR-ABL, collectively indicating that KDM6A fulfils an intrinsic survival role for CML cells upon inhibition of BCR-ABL signaling. Nevertheless, KDM6A expression was comparatively upregulated in imatinib-resistant versus imatinib-sensitive CML cells from patients including some patients displaying supra-physiological levels. How this occurs remains to be determined but to our knowledge there are only 0.15% KDM6A gene amplifications reported for the most common alterations in KDM6A 37.

An important finding of this study was that KDM6A protected CML cells against imatinib independently of its demethylase activity. This was firstly suggested by the inability of GSK-J4, which inhibits the demethylase activity of KDM6A, to reduce the sensitivity of CML cells to imatinib, and was further substantiated by the observation that an enzyme-dead KDM6A mutant, similar to wild-type KDM6A, recovered the resistance to imatinib in CML cells with endogenous KDM6A knocked out. Although the biological functions of KDM6A are primarily attributable to its nature as a demethylase 38, demethylase activity-independent roles of KDM6A are emerging 14, 39, 40. For example, demethylase-independent mechanisms were responsible for KDM6A-mediated regulation of gene expression during mouse embryonic development 11, whereas KDM6A suppressed squamous-like pancreatic cancer growth in male mice similarly independently of its demethylase activity 41. Our results have expanded the list of demethylase-independent functions of KDM6A by showing that its demethylase activity is dispensable for protection of CML cells against imatinib.

How does KDM6A execute its protective role against imatinib in CML cells? Our results revealed that this was achieved by transcriptional activation of the NGF receptor TRKA. Activation of pro-survival signaling by the NGF/TRKA axis has previously been associated with treatment resistance in hematological malignancies 42-44 lending support to the idea of employing TRKA inhibitors. Notably, pan-pharmacological inhibition of TRKs enhanced sensitivity of diffuse large B-cell lymphoma (DLBCL) to the monoclonal antibody against CD20 rituximab 45, whereas treatment with NGF protected TRKA-transformed myeloid 32D cells against irradiation 46. Of particular interest, NGF/TRKA signaling has been shown to be constitutively active and play an important role against imatinib through activation of the Akt pathway in BCR-ABL positive CML cells 23. It would be interesting to further identify the levels of TRKA and downstream signaling pathways of NGF before and after imatinib treatment in CML-sensitive and CML-resistant patients. Recently it has been suggested that TRKA inhibitors could be useful in targeting CML cells bearing oncogenic NTRK-fusion proteins 44. TRKA inhibitors are not presently indicated for CML treatment nor other hematological malignancies but our findings implore further consideration of this concept. Indeed the correlation between KDM6A and TRKA mRNA expression in patient-derived samples and demonstration of their functional dependency in *ex vivo* CML cells further corroborates the importance of KDM6A in regulation of TRKA expression in CML cells.

Additionally, we found that YY1 is responsible for transcriptional upregulation of TRKA by KDM6A. Notably, YY1-mediated histone modifications (acetylation and deacetylation) are involved in its roles in repressing and activating promoters 47, 48. We showed that YY1 interacts with KDM6A and appears to be responsible for recruiting KDM6A to the NTRK1 enhancer site. Furthermore, since the expression of KDM6A was required to maintain histone acetylation at the *NTRK1* enhancer site, it can be speculated that YY1 maintains this chromatin region in a permissive epigenetic state as a result of its interaction with KDM6A. Irrespectively, our results indicate that YY1-mediated activation of TRKA plays an important role in promoting resistance of CML against TKIs through the NGF/TRKA axis. These findings, together with previous results showing that YY1 is upregulated in CML 49, points to an oncogenic role of YY1 in CML.

In conclusion, we have provided evidence that KDM6A-regulated, YY1-mediated transcriptional upregulation of TRKA is an important resistance mechanism against imatinib in CML. The practical relevance of our results was corroborated by the findings that KDM6A together with TRKA were upregulated in patient-derived CML cells, and in particular, KDM6A was further increased in CML samples from imatinib-resistant individuals. Moreover, our results demonstrated that NGF-induced resistance to imatinib was abandoned in CML cells deficient of KDM6A. Further preclinical evaluation of targeting KDM6A as a strategy to overcome resistance of CML to TKIs is therefore warranted. Similarly, further details of how KDM6A is upregulated in CML cells need to be determined.

## Materials and Methods

### Antibodies

Antibodies used for this study were directed against phospho-BCR-ABL (210 kDa, 3901S), H3K27me3 (17 kDa, 9733), PARP (89 & 116 kDa), Akt (60 kDa, 9272S), KDM6A (155 kDa, 33510) from CST (Danvers, MA, USA); BCR-ABL (210 kDa, ab187831), TRKA (145 kDa, ab8871), H3K27ac (17 kDa, ab4729) from abcam (MA, USA); Flag (T0003), YY1 (70 kDa, 22156-1-AP), beta actin (42 kDa, 20536-1-AP), c-FOS (60 kDa, 66590-1-lg), CBP (29 kDa, 11149-1-AP) from Proteintech, IL, USA; KDM6A (155 kDa, A302-374A, Bethyl Laboratories, Montgomery, Texas, USA); phospho-AKT (60 kDa, GTX128414, GeneTex, CA, USA); phospho-Erk (44 kDa, 11245), Erk (42/44 kDa, 44204) from SAB, Maryland, USA; GAPDH (36 kDa, AP0063, Bioworld, Nanjing, China); RNA pol II (217 kDa, 39497) from Active Motif, Carlsbad, CA.

### CML patients

Archival bone marrow samples from diagnostic, imatinib-sensitive or resistant CML patients along with ITP (idiopathic thrombocytopenic purpura) control samples were obtained from The Affiliated Huaian first People's Hospital of Nanjing Medical University (Huai'an, China). The study was approved by the Institutional Ethics Committee of the hospital. All samples were obtained under written (signed) informed consent. The clinical and demographic details of all patients including those who all received imatinib therapy and were divided into subgroups according to their clinical response (sensitive versus resistant) are shown in Table S1.

### Cell culture and isolation of mononuclear cells

The K562, K562/G01 50, 51, MEG-01 and KU812 CML cell lines used are described in Table S2. K562 KDM6A KO cell lines were generated by CRISPR/Cas9 system according to Zhang's protocol 52. All CML cells were cultured in RPMI 1640 medium supplemented with 10% FBS at 37 °C in 5% CO2. U2OS cells were obtained from the Hong laboratory (Southeast University, China) and cultured in DMEM supplemented with 10% FBS. Mononuclear cells were isolated from fresh bone marrow isolates using Ficoll-Paque media (GE 17-5442-02) according to the manufacturers recommended protocol before culture in StemSpanTM SFEM II serum-free medium (STEMCELL, 09655, Canada) with addition of four growth factors, 20 ng/mL interleukin 3 [IL-3], 20 ng/mL interleukin 6 [IL-6],100 ng/mL Flt3-ligand and 20 ng/mL granulocyte colony-stimulating factor [G-CSF]( PeproTech).

### Vector construction

Individual sgRNA sequences (Table S3) were cloned into LentiCRISPRV2 vector (Addgene Plasmid 52961). cDNAs encoding KDM6A wild-type, KDM6A enzyme dead mutant and TRKA were cloned into the pLenti-EFs-FLAG-BSD lentiviral vector with a blasticidin gene for N-terminal tagging with FLAG epitope. KDM6A enzyme dead mutant was generated using QuikChange Lightning Site-Directed Mutagenesis Kit (Agilent technologies). ShRNAs against all different KDMs were cloned into lentivirus shRNA expression plasmid pLVshRNA-puro (Inovogen Tech. Co., Beijing, China). The primer pairs used for cloning are described in Table S3. Cas9 resistant construct of KDM6A was generated by mutating the PAM site to render it resistant to CRISPR/Cas9 without changing amino acids (KDM6A sgRNA2: CCTGCAGCGAAACGCACTCACTC → TTTGCAGCGAAACGCACTCACTC).

### Lentiviral transduction and clone derivation

CRISPR-CAS9 lentiviral particles were produced in 293FT cells after transfecting cells with a 2:1:2 mixture of psPAX2, pCMV-VSV-G and pLentiCrispr V2 plasmids using the Lipofectamine 2000 Reagent (Thermo) according to the manufacturers instructions. Alternatively for knockdown or overexpression studies, the pLVshRNA-puro or pLenti-EFs-FLAG-BSD-based vectors were substituted as required. Supernatants containing the viral particles were harvested at 48 h and centrifuged at 500 g for 5 min before filtering through a 0.45 μm PES filter. To increase viral titres the supernatants were precipitated by adding 4 ℃ PEG Virus Precipitation Solution (ab102538, abcam, MA, USA) at a 1:4 v/v ratio, refrigerating overnight before centrifuging the PEG mixture at 1500 × g for 30 min at 4 ℃. The precipitated lentiviral particles were resuspended in PBS and added to the culture medium for 36 h before selecting with 1 μg/mL puromycin (pLentiCrispr V2) or 10 μg/mL blasticidin (pLenti-EFs-FLAG-BSD). Selected cells were single cell cloned in 96 well plates and genomic DNA was amplified using the screening primers listed in Table S3. PCR products were TA-cloned and at least six fragments subjected to Sanger sequencing for each clone. Western blot analysis was then used to verify knockout or overexpression. For the fresh bone marrow samples, isolated mononuclear cells were cultured for 24 h before the addition of shRNA lentivirus for a further 24 h and selected in 1 μg/mL puromycin for a further 48 h.

### Real-Time quantitative Polymerase Chain Reaction (qPCR)

Total RNAs were isolated using RNeasy Kits (Qiagen, 74104, Hilden, Germany) according to the manufacturer's instructions. cDNA was synthesized using the Primescript RT-reagent kit (Takara, RR047A, Shiga, Japan) containing gDNA eraser (DNAse) according to the manufacturer's instructions. The qPCR reactions were performed using the LightCycler 480 (Roche, Basel, Switzerland) with relative expression determined using the ΔΔCt method with normalization against β-actin. The primers which span exon-exon junctions to prevent gDNA signal are provided in Table S3.

### Histone extraction

Histones were acid extracted as previously described 53. Briefly, 2×10^6^ cells were collected and resuspended in 200 μL NETN lysis buffer (20 mM Tris-HCl pH 8.0, 500 mM NaCl, 0.5% NP-40 (v/v), 1 mM EDTA, 2 mM phenylmethylsulfonyl fluoride (PMSF)) at 4 °C. After centrifugation, the pellet was resuspended in 0.2 N HCl at a cell density of 2×10^7^ cells per mL. Histones were extracted by rotating at 4 °C overnight and then the protein concentration was determined using the BCA kit.

### Western blotting

Total cell extracts were prepared in lysis buffer containing 2% SDS, 62.5 mM Tris pH 6.8, 10% glycerol, 5% β-mercaptoethanol. Protein samples were heated at 95 °C for 10 min and analyzed by SDS-PAGE on 8% or 12% gels (45 min, 200 V) (Bio-rad Laboratories), followed by immunoblotting on PVDF membranes using a wet blotting system (70 min, 250 mA) (BioRad Laboratories). Membranes were incubated with primary and secondary antibody diluted in 5% milk/TBST solutions, Western blots were developed using Western Bright peroxide (Advansta, 180129-34, CA, USA) and visualized on ChemiDoc XRS+ system (Bio-Rad, CA, USA).

### Immunofluorescence by confocal microscopy

K562 cells were centrifuged on glass coverslips, fixed with 4% paraformaldehyde in PBS for 20 min, and subsequently permeabilized with 0.1% Triton X-100 for 10 min. After blocking with 1% BSA for 1 h at room temperature , cells were incubated with primary antibody overnight at 4 °C, followed by secondary antibody conjugated with CoraLite488 (SA00013-1, Pro-teintech, IL, USA) for 1 h at room temperature. After three washes with PBS, cells were mounted with Aqueous Mounting Medium containing 4',6-diamidino-2-phenylindole (DAPI, Beyotime, C1005, Shanghai, China) and visualized with a confocal microscope.

### Apoptosis assay

Analyses were performed using Annexin V-FITC/PI apoptosis detection kit (KeyGen, KGA107, Nanjing, China) according to the manufacturer's protocol. Briefly, CML cells were seeded into 12-well plates with 2.5 × 10^5^ each well and treated with 0.5 µM imatinib and/or 100 ng/mL NGF for 24 h. Cells were then harvested and washed with PBS for three times before addition of 500 μL binding buffer, 5 μL Annexin V-FITC and 5 μL PI solution to the cell pellet for 15 min at room temperature in the dark before flow cytometric analysis to detect early apoptotic cells (Annexin-V positive and PI-negative) (Beckman Coulter, USA).

### RNA-Seq analysis

Transcriptome analyses were performed comparing the control K562 or K562/G01 cells against the KDM6A deletion group as indicated. RNA-Seq was performed using an Illumina Hiseq platform (Novogene, Beijing, China). Differential expression analysis of two groups was performed using the ballgrown R package (1.16.1). Ballgown provides statistical routines for determining differential expression in digital gene expression data using a model based on the negative binomial distribution 54. The resulting *p*-values were adjusted using the Benjamini and Hochberg's approaches to control false discovery rate. Genes with an adjusted *p*-value <0.05 were considered to be differentially expressed. The clusterProfiler R package was used to detect the statistical enrichment of differential expression genes in KEGG pathways (http://www.genome.jp/kegg/). The raw data of RNA-seq was deposited in the NCBI Sequence Read Archive (SRA) under accession numbers, SRR 11452004-SRR11452009 and SRR12755782-SRR12755787.

### Luciferase assay

Empty pGL3-promoter or *NTRK1*-containing luciferase reporter plasmids were co-transfected with pRL-TK into U2OS cells along with control vector, KDM6A or the KDM6A-ED mutant as indicated. Luciferase activity after 48 h measured by the Dual Luciferase Reporter Gene Assay Kit (Beyotime, RG027, Shanghai, China) on the Hidex Sense instrument (Hidex, Finland). Firefly luciferase readings were normalized to the co-transfected Renilla Luciferase control.

### Chromatin immunoprecipitation assay

Chromatin immunoprecipitation (ChIP) assays were performed using the Millipore ChIP kit according to the manufacturer's protocol 55. Briefly, 3×10^7^ cells were collected, fixed and sonicated with a Bioruptor sonicator (Diagenode) to generate DNA fragments of approximately 500 bp in length. Chromatin immunoprecipitates for proteins were amplified by quantitative PCR, normalized to input, and calculated as percentages of inputs. Fold enrichment levels indicate the fold changes over the negative control immunoglobulin G (IgG). The PCR primer sequences are described in Table S3.

### Cell viability assay

Cells were grown in medium containing 10% FBS for 24/72 h in the absence or presence of β-NGF (PeproTech, 450-01, Suzhou, China) and/or imatinib (MCE, HY-15463, NJ, USA). The number of living cells was counted on Countless II FL (Life technology) using 0.4% Trypan Blue.

### Immunoprecipitation

Cells were extracted using the IP lysis buffer (Pierce, 87787, IL, USA) plus EDTA free protease inhibitor cocktail and PMSF. The clarified cell lysates (1×10^7^ cells) were incubated at 4 ℃ for 12 h with anti-KDM6A antibody preabsorbed on Protein A/G Magnetic Beads (Pierce, 88803, IL, USA). After washing, the immunoprecipitates were denatured and subjected to SDS-PAGE and Western blot analyses with the appropriate antibodies.

### Statistical analysis of data

SPSS software version 19.0 (IBM Corp., Armonk, New York, US) and GraphPad Prism v6 (GraphPad Software, Inc., San Diego, California, US) were used for all statistical analyses. Data were first evaluated for normal distribution using the Shapiro-Wilk method and homogeneity of variance with the Levene method. Pairwise comparisons of normally distributed data were analysed using Student's t test or for multigroup comparisons, one-way analysis of variance (ANOVA) with post hoc Tukey's test. Data not meeting normal distribution/homogeneity of variance were compared using Kruskal-Wallis and Mann-Whitney non-parametric tests. *, *p* < 0.05; **, *p* < 0.01; and ***,* p* < 0.001 denote statistically significant changes.

## Supplementary Material

Supplementary figures and tables.

## Abbreviations

ALL: acute lymphoblastic leukemia
AML: acute myelogenous leukemia
ChIP: chromatin immunoprecipitation
CMML: chronic myelomonocytic leukemia
CML: chronic myelogenous leukemia
Co-IP: Co-immunoprecipitation
COMPASS: complex of proteins associated with Set1
DEGs: differentially regulated genes
DLBCL: diffuse large B-cell lymphoma
IgG: immunoglobulin G
ITP: idiopathic thrombocytopenic purpura
JmjC: Jumanji
KDM6A-ED: enzymatically-dead KDM6A mutant
KDMs: histone lysine demethylases
MF: middle fragment
NGF: nerve growth factor
PBMCs: peripheral blood mononuclear cells
PMSF: phenylmethylsulfonyl fluoride
Ph: Philadelphia
TKIs: tyrosine kinase inhibitors
TPR: tetratricopeptide repeat
TRKA: tropomyosin receptor kinase A
TSS: transcription start site
