# Supplementary material for: KDM6A promotes imatinib resistance through YY1-mediated transcriptional upregulation of TRKA independently of its demethylase activity in chronic myelogenous leukemia
Source: Theranostics. 2021 Jan 1;11(6):2691–705. doi: 10.7150/thno.50571 (PMC7806474; doi:10.7150/thno.50571)
Supplement: Supplementary file 1 — Supplementary figures and tables. [file thnov11p2691s1.pdf]

Supplementary Materials

KDM6A promotes imatinib resistance through YY1-mediated transcriptional upregulation of TRKA independently of its demethylase activity in chronic myelogenous leukemia

Chengwan Zhang[1], Li Shen[2], Yifu Zhu[4], Ran Xu[3], Zhikui Deng[2], Xiaoning Liu[2], Yihan Ding[2], Chunling Wang[2], Yuye Shi[2], Liye Bei[2], Dongping Wei[5], Rick F. Thorne[6,7], Xu Dong Zhang[6,8], Liang Yu[1,2]*, Song Chen[3,6]*

[1]Department of Central Laboratory, The Affiliated Huaian No. 1 People's Hospital, Nanjing Medical University, Huai'an, Jiangsu, 223300, China

[2]Department of Hematology, The Affiliated Huaian No.1 People's Hospital,Nanjing Medical University, Huai'an, Jiangsu, 223300, China

[3]Institute of Medicinal Biotechnology, Jiangsu College of Nursing, Huai'an, Jiangsu, 223300, China

[4]The Chinese Academy of Sciences (CAS), Key Laboratory of Innate Immunity & Chronic Disease, CAS Center for Excellence in Cell & Molecular Biology, School of Life Sciences, University of Science & Technology of China, Hefei 230026, China

[5]Department of Oncology, Nanjing First Hospital, Nanjing Medical University, Nanjing, 210006, China

[6]Translational Research Institute of Henan Provincial People's Hospital and People's Hospital of Zhengzhou University, and Molecular Pathology Center, Academy of Medical Sciences, Zhengzhou University, Zhengzhou, Henan 450053, China

[7]School of Environmental and Life Sciences, The University of Newcastle, NSW, 2258, Australia

[8]School of Biomedical Sciences and Pharmacy, The University of Newcastle, NSW, 2308, Australia

* To whom correspondence may be addressed. Email: hayyyl@njmu.edu.cn (L.Y) or schen@zzu.edu.cn (S.C)

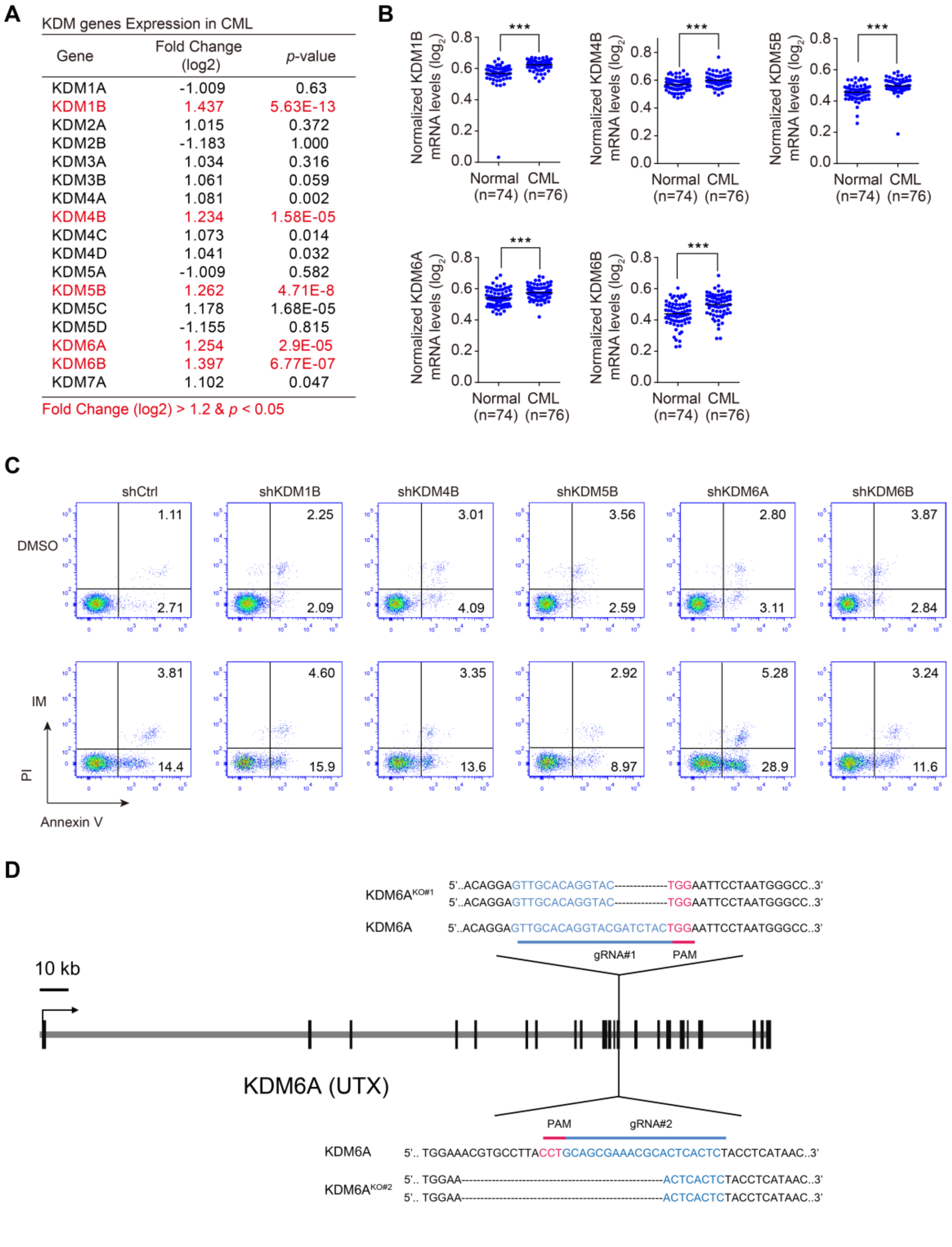

Figure S1. Differential expression of KDMs in CML and CRISPR-mediated knockout of KDM6A.

(A, B) Relative mRNA expression changes amongst KDM family members between non-leukemia and healthy bone marrow (n=74) and CML (n=76) derived from the oncomine Haferlach Leukemia dataset (GEO accession no.13159) **(A)**. Five KDMs with $\log_2$fold change > 1.2 and *p* values < 0.05 are highlighted in red and data derived from GEO2R online

analysis of the relative expression levels of KDMs in gene expression dataset, GSE13159 were plotted in **(B)** using GraphPad Prism 6. Horizontal bar shows mean. Unpaired, two-tailed Student's t-test; Mann-Whitney test (KDM1B, KDM5B); ***$p < 0.001$.

(C) Representative FACS dotplots of FITC Annexin V/PI staining used to assess apoptosis after shRNA-mediated knockdown of different KDMs in the K562 cell line. Treatment responses comparing cells treated with vehicle (DMSO) or 0.5 μM IM for 24 h.

(D) Schematic illustration of the KDM6A gene structure and genomic sequences of two K562 KDM6A knockout clones generated *via* the CRISPR/Cas9 system. PAM sequences and gRNA targeted sequences are highlighted in pink and blue, respectively, with deleted sequences designated by dashes.

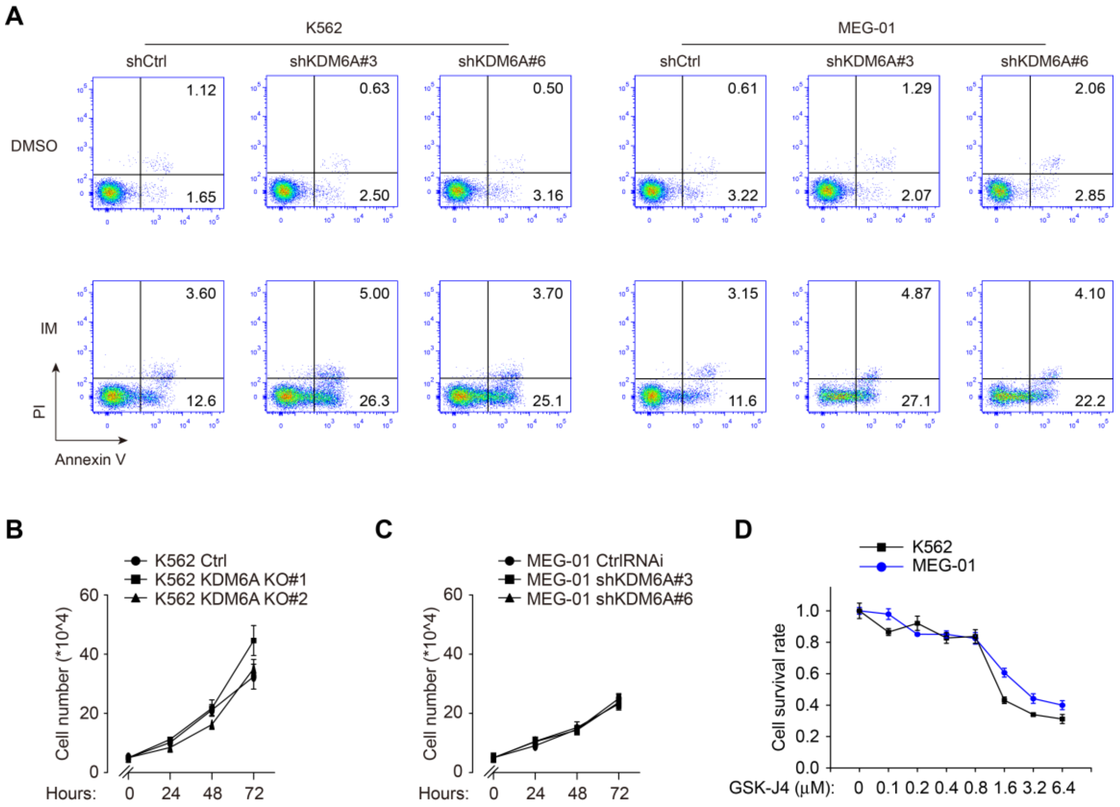

Figure S2. Role of KDM6A in imatinib-induced apoptosis in K562 and MEG-01 cell lines.

(A) Representative FACS dotplots of FITC Annexin V/PI staining used to assess apoptosis after knockdown of KDM6A using two independent shRNAs targeting KDM6A in the K562 and MEG-01 cell lines. Treatment responses comparing cells treated with vehicle (DMSO) or 0.5 µM IM for 24 h.

(B,C) KDM6A depletion has no effect on K562 (B) and MEG-01 (C) cell proliferation. (B) The KDM6A KO#1 and KO#2 K562 cells and their control cells were seeded.(C) and then the cell number was counted by trypan blue exclusion method at the times indicated. The results are shown as mean ±s.d. of triplicated cultures.

(D) GSK-J4 dose-response effects on cell viability were determined in CML cell lines after 24 h treatment using CCK-8 assay. Mean ±s.d. are given for three independent experiments.

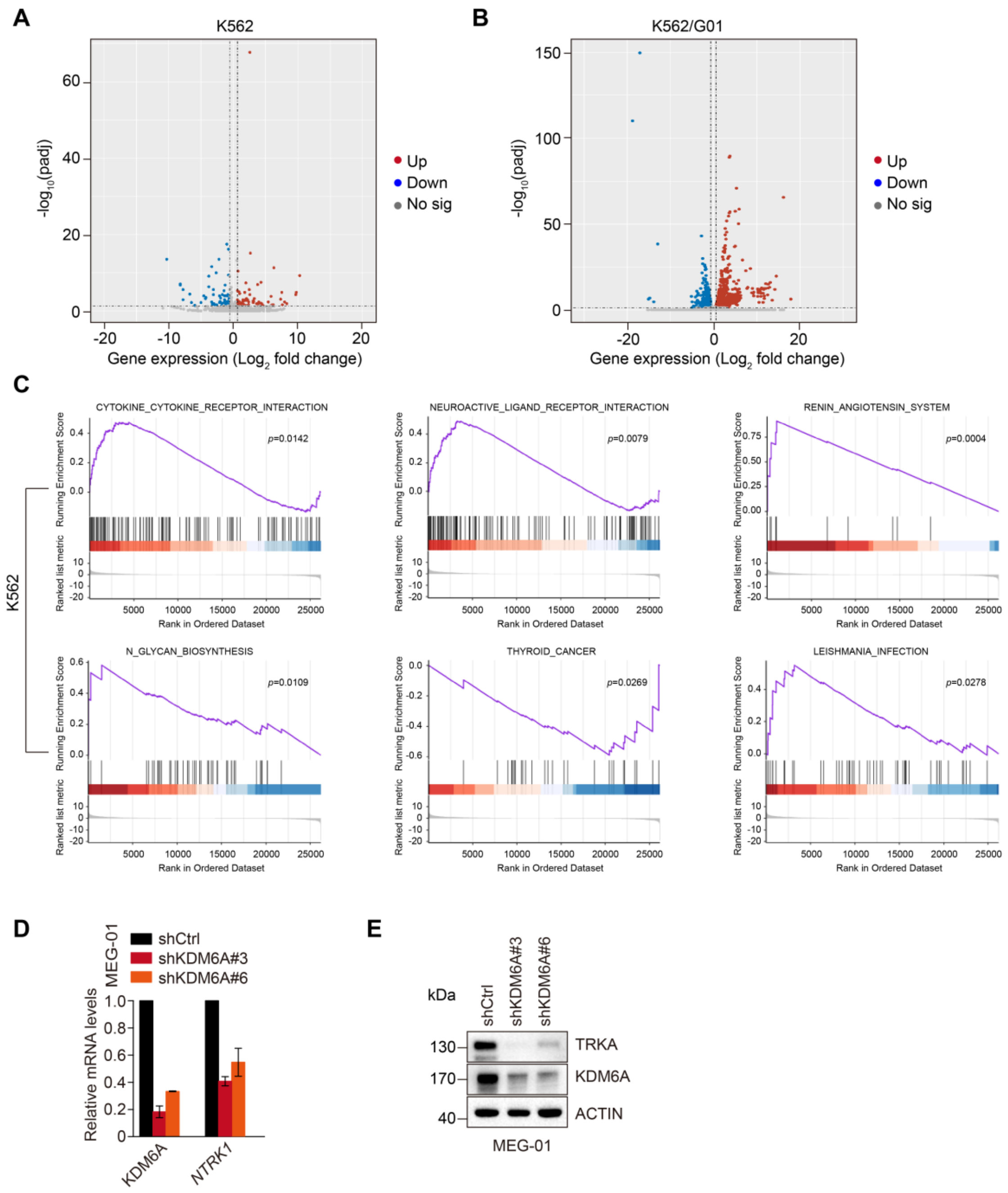

Figure S3. Differentially expressed genes following loss of KDM6A.

(A,B) Genes differentially expressed between control and KDM6A-depleted K562 or K562/G01 cells using RNAseq. Volcano plot shows the $\log_2$ fold change (x-axis) and significance (−log10* adjusted p-value; y-axis) with significantly downregulated and upregulated genes shown in blue and red, respectively. Significance cutoffs for differential expression were adjusted $p < 0.05$ and $|\log2foldchange| > 0.6$.

(C) KEGG pathway analysis of K562 RNA-seq data using GSEA.

(D, E) Analysis of TRKA expression after depletion of KDM6A in MEG-01 cells using qPCR **(D)** and Western blot **(E)**. Mean ± s.d. are given for three independent experiments.

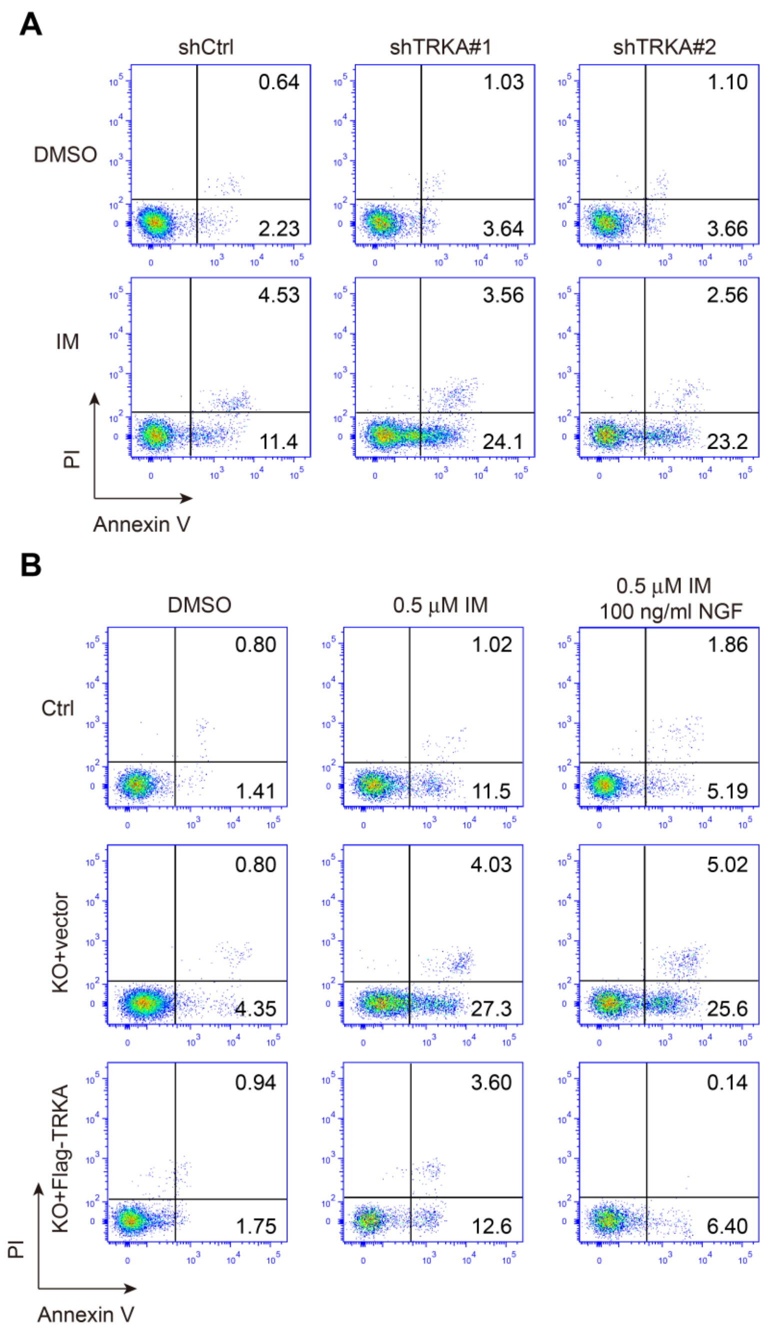

Figure S4. Role of TRKA and NGF signaling in CML cell line resistance to imatinib

(A) Representative FACS dotplots of FITC Annexin V/PI staining used to assess apoptosis after shRNA-mediated knockdown using two independent shRNAs targeting TRKA in the K562 cells treated with vehicle (DMSO) or 0.5 μM IM for 24 h.

(B) Representative FACS dotplots of FITC Annexin V/PI staining used to assess apoptosis after addition of carrier (DMSO) or 0.5 μM imatinib for 24 h alone or in combination with 100 ng/mL NGF. Parental control K562 cells are compared with KDM6A KO cells transfected with control (vector) or Flag-TRKA.

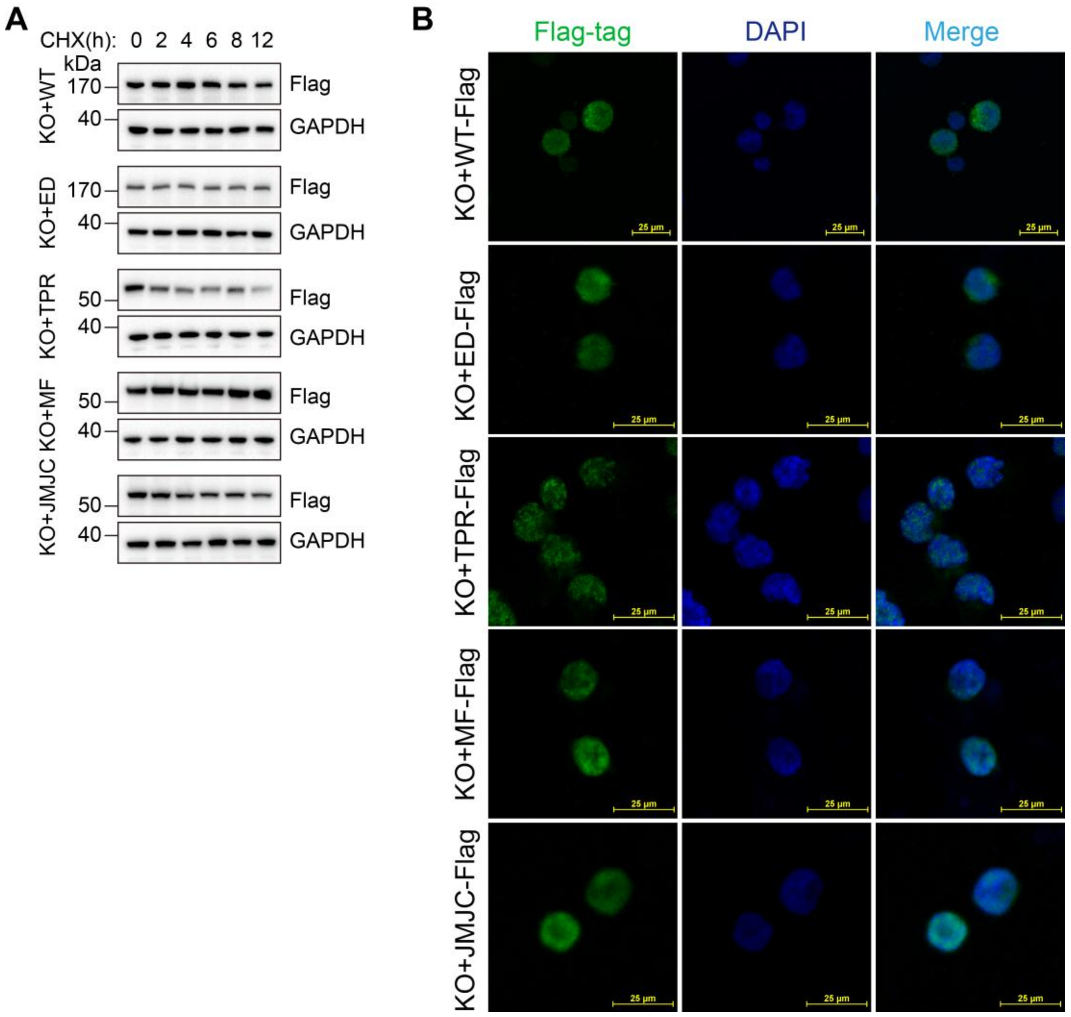

Figure S5. The stability and localization of KMD6A mutants

(A) Half-life of wildtype KDM6A and truncation mutants in K562 cells was assessed by cycloheximide (CHX) treatment for indicated times.

(B) The localization of KDM6A protein and truncations in K562 cells by immunofluorescence microscopy.

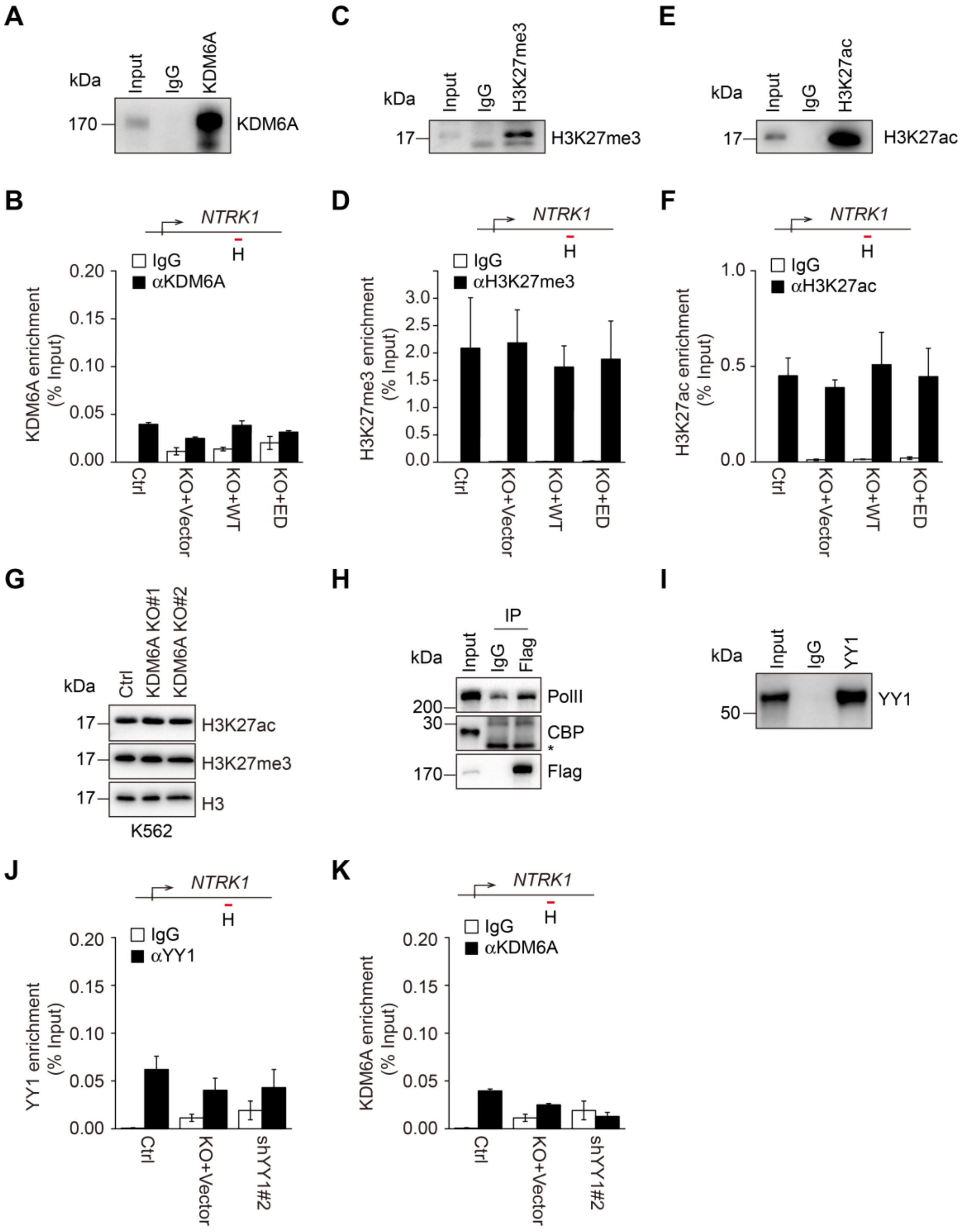

Figure S6. KDM6A is recruited to the NTRK1 promoter by YY1

(A-F) K562 cells were subjected to CHIP assay using antibodies against KDM6A **(A)**, H3K27me3 **(C)** and H3K27ac **(E)**. Western blot showing the efficient enrichment of targets as indicated **(A,C,E)**. IgG was used as a control.

ChIP-qPCR assays were conducted as per (Fig4.I) against KDM6A **(B)**, H3K27me3 **(D)**, H3K27ac **(F)** against amplicon targeting H, comparing parental K562 cells (Ctrl) against

KDM6A KO#2 cells reconstituted with vector (KO+vector), KDM6A (KO+WT) or KDM6A-ED (KO+ED). No enrichment was observed on amplicon H, used as a negative control. Mean ± s.d. are given for three independent experiments.

(G) Total levels of H3K27me3 and H3K27ac from K562 cell lines as indicated were determined by western blot. Total histone H3 was used as a loading control.

(H) Immunoprecipitation showing Flag-KDM6A binds to RNA polII but not CBP in K562 cells. IgG was used as a control.

(I) K562 cells were subjected to CHIP assay using antibodies against YY1 antibody to comfirm antibody binding efficiency. IgG was used as a control.

(J, K) ChIP-qPCR assays were conducted using antibodies against YY1 **(I)** or KDM6A **(J)** in control K562 versus KDM6A-KO cells or cells with YY1 knockdown. Mean ± s.d. are given for three independent experiments.

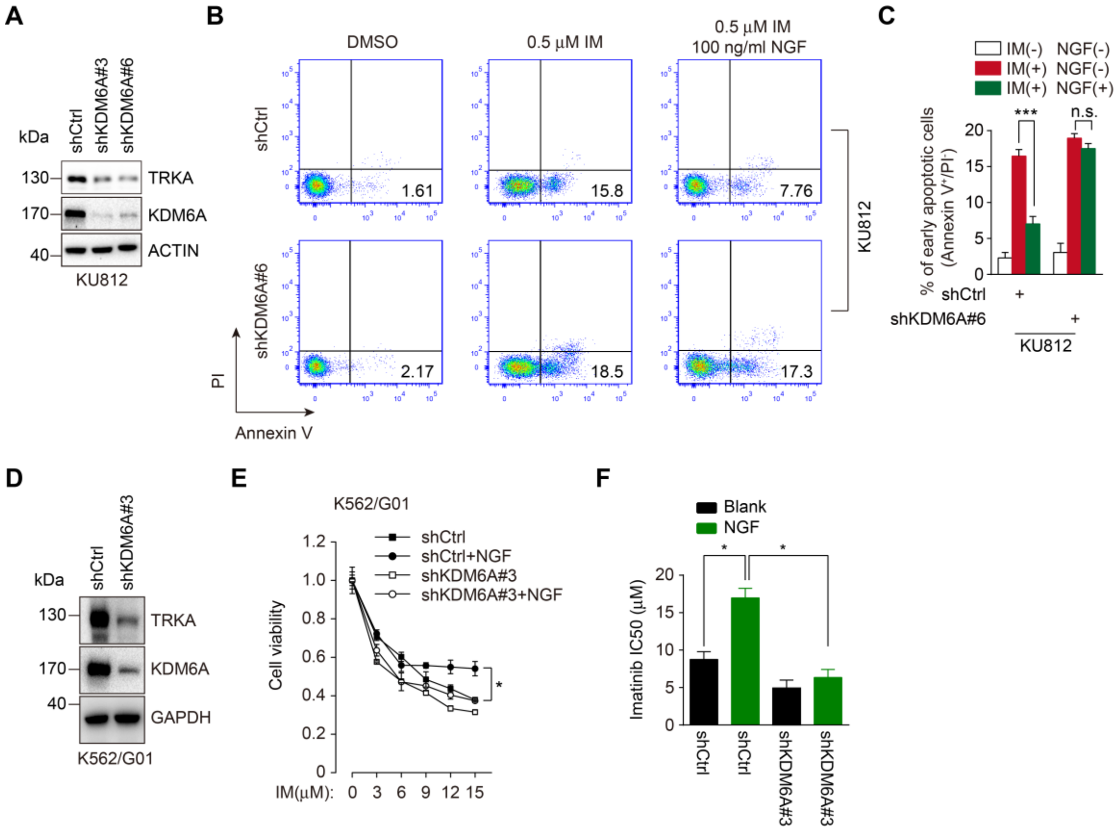

Figure S7. Role of TRKA and NGF signaling in CML cell line resistance to imatinib

(A) Western blot verification of KDM6A RNAi efficiency and effect on TRKA expression in KU812 cells.

(B) Representative FACS dotplots of FITC Annexin V/PI staining used to assess apoptosis after addition of carrier (DMSO) or 0.5 μM imatinib for 24 h alone or in combination with 100 ng/mL NGF. Parental control KU812 cells are compared with KDM6A KD cells.

(C) The rates of apoptosis in shCtrl versus shKDM6A KU812 cells after 24 h treatment with DMSO vehicle control or 0.5 μM imatinib alone or in combination with 100 ng/mL NGF. Unpaired, two-tailed Student's t-test; ***$p < 0.001$; n.s., not significant.

(D) Western blot verification of KDM6A RNAi efficiency in imatinib-resistant K562/G01 cells.

(E, F) Cell survival comparisons between of K562/G01 control and KDM6A RNAi after 24 h of growth in 10% FBS containing medium supplemented with or without 100 ng/mL NGF in the presence of imatinib (dose indicated on the abscissa) **(E)**. Live cells were counted by Trypan blue exclusion and results calculated as a percentage of growth seen with untreated controls. Comparison of IC50 values for imatinib with or without 100 ng/mL NGF in K562/G01 cell lines **(F)**. The IC50 values were determined using GraphPad Prism. Mean ± s.d. are given for three independent experiments. Mean ± s.d. are given for three independent

experiments. One-way ANOVA; *p <0.05.

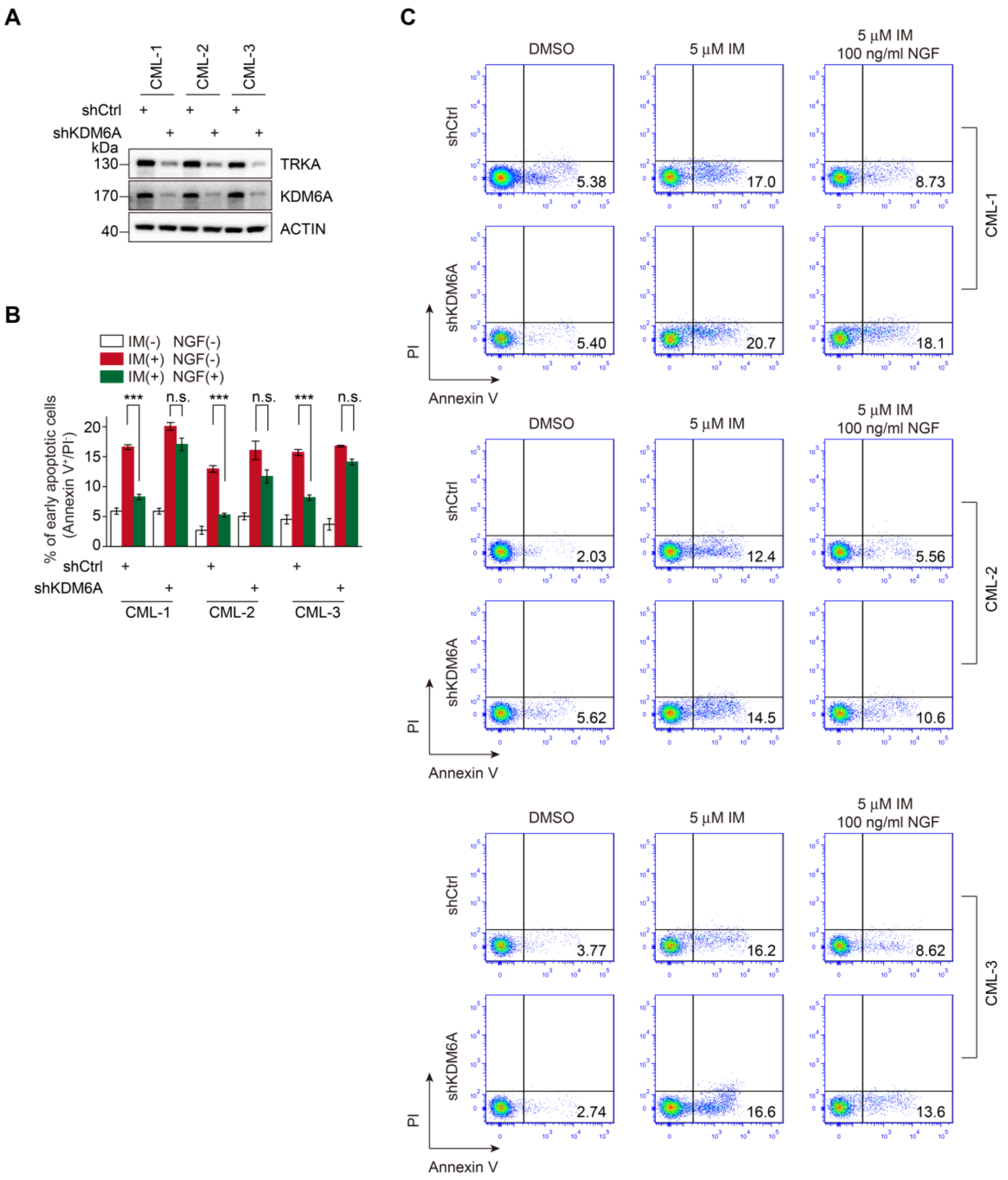

Figure S8. Role of TRKA and NGF signaling in imatinib resistance of *ex vivo* CML cells

(A) Western blot verification of KDM6A RNAi efficiency and effect on TRKA expression in CML patients cells.

(B) The rates of apoptosis in shCtrl versus shKDM6A CML patients cells after 48 h treatment with DMSO vehicle control or 5 μM imatinib alone or in combination with 100 ng/mL NGF. The percentage of early apoptotic cells was determined by FACS using annexin V/PI double staining. Mean ± s.d. are given for three independent experiments. Unpaired, two-tailed Stu-

dent's t-test; ***p < 0.001.

(C) Representative FACS dotplots of FITC Annexin V/PI staining used to assess apoptosis after addition of carrier (DMSO) or 5 μM imatinib for 48 h alone or in combination with 100 ng/mL NGF. Parental control CML patients cells are compared with KDM6A KD cells.

Table S1 Patient cohort and demographics

No.	Group	Type of disease	Age	Gender	Stage	Therapy	BCR/ABL	Blast count (%)	KDM6A expression	NTRK1 expression
C01	Ctrl	ITP	70	Male	Not applicable	Not applicable	Not applicable	1.00	1.18	0.28
C02	Ctrl	ITP	37	Female	Not applicable	Not applicable	Not applicable	0.50	0.83	0.29
C03	Ctrl	ITP	37	Female	Not applicable	Not applicable	Not applicable	1.50	0.96	2.73
C04	Ctrl	ITP	36	Female	Not applicable	Not applicable	Not applicable	0.00	1.17	2.90
C05	Ctrl	ITP	48	Male	Not applicable	Not applicable	Not applicable	0.00	0.61	1.36
C06	Ctrl	ITP	55	Female	Not applicable	Not applicable	Not applicable	0.50	1.50	1.15
ND01	ND	CML	51	Male	AP	Not applicable	positive	14.00	1.98	5.09
ND02	ND	CML	57	Male	CP	Not applicable	positive	1.50	1.27	6.23
ND03	ND	CML	42	Female	CP	Not applicable	positive	0.00	1.87	2.84
ND04	ND	CML	25	Female	CP	Not applicable	positive	2.00	1.62	5.65
ND05	ND	CML	43	Female	CP	Not applicable	positive	3.00	1.04	4.88
ND06	ND	CML	40	Female	CP	Not applicable	positive	1.00	1.49	1.76
ND07	ND	CML	24	Male	CP	Not applicable	positive	1.50	2.98	8.05
ND08	ND	CML	33	Male	CP	Not applicable	positive	0.00	1.10	1.66
ND09	ND	CML	70	Male	CP	Not applicable	positive	1.00	1.37	4.28
ND10	ND	CML	29	Female	CP	Not applicable	positive	1.50	2.80	6.96
ND11	ND	CML	43	Female	CP	Not applicable	positive	1.00	1.66	6.23
ND12	ND	CML	51	Male	CP	Not applicable	positive	2.00	2.65	9.57
S01	IM-S	CML	49	Male	CP	Imatinib	positive	2.50	0.55	2.73
S02	IM-S	CML	65	Male	CP	Imatinib	positive	2.00	3.48	3.26
S03	IM-S	CML	53	Male	CP	Hydroxyurea, Imatinib	positive	0.50	0.69	0.04
S04	IM-S	CML	46	Female	CP	Imatinib	positive	1.50	1.01	0.00

S05	IM-S	CML	51	Female	AP	Hydroxyurea, Imatinib	positive	13.00	3.55	0.01
S06	IM-S	CML	54	Male	AP	Imatinib	positive	15.00	0.60	0.03
S07	IM-S	CML	51	Male	BP	Imatinib	positive	39.00	0.72	0.02
S08	IM-S	CML	51	Female	CP	Imatinib	positive	0.50	1.47	3.55
S09	IM-S	CML	39	Male	CP	Imatinib	Undetermined	1.50	1.60	2.36
S10	IM-S	CML	49	Female	CP	Imatinib	Undetermined	0.00	1.62	0.48
S11	IM-S	CML	17	Male	CP	Imatinib	positive	0.50	1.09	6.31
S12	IM-S	CML	69	Female	CP	Imatinib	positive	1.50	2.20	4.49
S13	IM-S	CML	62	Male	BP	Imatinib	positive	90.50	3.27	0.04
S14	IM-S	CML	54	Male	CP	Cytarabine, Imatinib	Undetermined	1.00	0.96	3.18
S15	IM-S	CML	50	Female	CP	Imatinib	positive	1.00	0.78	9.04
S16	IM-S	CML	71	Female	CP	Imatinib	positive	1.00	0.50	7.25
S17	IM-S	CML	44	Male	BP	Imatinib	positive	23.50	0.03	1.07
S18	IM-S	CML	19	Male	CP	Imatinib	positive	1.00	0.32	6.31
S19	IM-S	CML	51	Male	CP	Imatinib	positive	3.50	0.96	0.51
S20	IM-S	CML	53	Female	CP	Imatinib	positive	0.50	1.34	4.65
S21	IM-S	CML	52	Female	CP	Imatinib	positive	1.50	3.34	1.49
S22	IM-S	CML	47	Male	CP	Hydroxyurea, Imatinib	positive	1.00	1.86	6.01
S23	IM-S	CML	6	Male	CP	Imatinib	positive	2.00	1.05	1.22
S24	IM-S	CML	33	Male	CP	Imatinib	positive	1.50	1.33	0.60
S25	IM-S	CML	35	Male	CP	Imatinib	positive	2.00	0.39	0.48
S26	IM-S	CML	57	Female	CP	Imatinib	positive	0.50	0.98	0.25
S27	IM-S	CML	61	Male	CP	Imatinib	positive	1.50	1.42	1.67
S28	IM-S	CML	58	Male	CP	Hydroxyurea, Imatinib	positive	1.00	0.81	5.04
S29	IM-S	CML	55	Male	BP	Hydroxyurea, Imatinib	Undetermined	29.50	0.59	0.11
S30	IM-S	CML	63	Male	CP	Imatinib	positive	1.50	3.93	0.17

S31	IM-S	CML	76	Female	CP	Hydroxyurea, Imatinib	positive	1.00	0.22	0.65
S32	IM-S	CML	43	Male	CP	Imatinib	Undetermined	1.00	2.40	4.51
S33	IM-S	CML	63	Male	CP	Imatinib	positive	0.50	0.70	0.36
S34	IM-S	CML	54	Male	CP	Imatinib	positive	2.50	1.28	0.53
S35	IM-S	CML	60	Female	BP	Imatinib	positive	75.50	0.46	1.79
S36	IM-S	CML	67	Male	CP	Imatinib	positive	1.50	1.91	1.95
S37	IM-S	CML	27	Female	CP	Imatinib	positive	0.50	1.14	0.88
S38	IM-S	CML	47	Female	CP	Imatinib	positive	0.50	2.65	5.43
S39	IM-S	CML	71	Male	CP	Imatinib	positive	0.50	0.69	1.03
S40	IM-S	CML	47	Male	CP	Imatinib	positive	0.50	1.98	5.09
S41	IM-S	CML	55	Male	CP	Imatinib	positive	1.00	1.27	6.23
S42	IM-S	CML	48	Female	CP	Imatinib	positive	1.00	1.87	2.84
S43	IM-S	CML	45	Male	BP	Imatinib	positive	23.50	1.62	5.65
S44	IM-S	CML	26	Female	CP	Imatinib	positive	0.50	1.04	4.88
S45	IM-S	CML	72	Male	CP	Imatinib	positive	1.00	1.49	1.76
S46	IM-S	CML	64	Male	CP	Imatinib	positive	1.00	2.98	8.05
S47	IM-S	CML	27	Female	CP	Imatinib	positive	0.00	1.10	1.66
S48	IM-S	CML	23	Male	CP	Imatinib	positive	0.50	1.37	4.28
S49	IM-S	CML	84	Male	CP	Imatinib	positive	0.50	2.80	6.96
S50	IM-S	CML	36	Female	CP	Imatinib	positive	0.00	1.66	6.23
S51	IM-S	CML	72	Male	CP	Imatinib	positive	2.00	2.65	9.57
R01	IM-R	CML	63	Male	AP	Imatinib, Homoharringtonine	positive	13.00	2.34	3.32
R02	IM-R	CML	27	Male	CP	Imatinib	positive	0.00	1.33	1.14
R03	IM-R	CML	61	Male	AP	Imatinib	positive	12.00	15.50	5.18
R04	IM-R	CML	50	Female	BP	Hydroxyurea,Imatinib,	positive	81.00	0.59	1.07

						Homoharringtonine				
R05	IM-R	CML	37	Male	CP	Imatinib	positive	2.50	1.58	1.40
R06	IM-R	CML	45	Female	CP	Imatinib	positive	2.00	1.18	0.90
R07	IM-R	CML	43	Male	CP	Imatinib	positive	1.00	0.68	1.28
R08	IM-R	CML	51	Female	AP	Imatinib	positive	10.50	9.54	7.27
R09	IM-R	CML	43	Female	CP	Imatinib	positive	0.00	3.24	0.12
R10	IM-R	CML	53	Male	CP	Imatinib	positive	1.00	4.11	3.43
R11	IM-R	CML	29	Male	CP	Imatinib	positive	1.00	0.72	7.20
R12	IM-R	CML	41	Female	CP	Imatinib	positive	0.50	1.38	7.15
R13	IM-R	CML	61	Male	CP	Imatinib	positive	0.50	2.44	0.09
R14	IM-R	CML	23	Male	CP	Imatinib	positive	1.50	0.42	1.14
R15	IM-R	CML	45	Male	BP	Imatinib	positive	25.00	16.20	38.51
R16	IM-R	CML	62	Male	CP	Imatinib	positive	0.00	0.04	23.54
R17	IM-R	CML	48	Female	AP	Imatinib	positive	15.00	2.27	10.26
R18	IM-R	CML	55	Male	BP	Imatinib	positive	36.00	4.07	7.40
R19	IM-R	CML	52	Female	CP	Imatinib	positive	0.00	8.13	22.45
R20	IM-R	CML	55	Female	CP	Hydroxyurea,Imatinib, Homoharringtonine	positive	1.00	2.37	9.44
R21	IM-R	CML	48	Male	CP	Imatinib	positive	0.50	9.08	59.23
CML-1	ND	CML	51	Female	CP	Not applicable	positive	1.50	N/A	N/A
CML-2	ND	CML	42	Female	CP	Not applicable	positive	2.00	N/A	N/A
CML-3	ND	CML	65	Male	CP	Not applicable	positive	1.00	N/A	N/A

N/A, not analyzed;Not applicable, patients were not subjected to treatment;AP, accelerated phase;BP, blastic phase;CP, chronic phase.

Patients in the chronic phase typically have less than 10% blasts in their blood or bone marrow samples.

Most patients with accelerated phase of CML have 10% to 19% blasts in both the blood and bone marrow.In the blast phase, there are 20% or more blasts in the blood or bone marrow.

Table S2 Cell lines used in the study

Cell line name	Cell Type	Gender	Mycoplasma Test	STR analysis
K562	CML in blast crisis	Female	Negative	Verified
K562/G01	CML in blast crisis	Female	Negative	Undetermined
MEG-01	CML in megakar-yoblastic crisis	Male	Negative	Verified
KU812	CML in blast crisis	Male	Negative	Verified

Table S3 Primer sequence information

**Usage**	**Name**		**Sequence**	**Tm (℃)**	**Amplicon length**	**Amplification efficiency(%)**
**shRNA**	control	Forward	GATCGGGTTCTCCGAACGTGTCAC-GTTTCCGAAGAAACGTGACACGTTCGGA-GAATTTTTC	73.3	Not applicable	Not applicable
		Reverse	AATTGAAAAATTCTCCGAACGTGTCAC-GTTTCTTCGGAAACGTGACACGTTCGGA-GAACCC	72.6		
	KDM1B	Forward	GATCGGGTCCCGGGTACTCGGTGA-TAATCGAAATTATCACCGAGTACCCGGGAT-TTTTC	72.6	Not applicable	Not applicable
		Reverse	AATTGAAAAATCCCGGGTACTCGGTGA-TAATTTCGATTATCACCGAGTACCCGGGACCC	71.9		
	KDM4B	Forward	GATCGGGGTGGAAGCTGAAATGCGTGTAC-GAATACACGCATTTCAGCTTCCACTTTTTC	72.5	Not applicable	Not applicable
		Reverse	AATTGAAAAAGTGGAAGCTGAAATGCGTG-TATTCGTACACGCATTTCAGCTTCCACCCC	71.8		
	KDM5B	Forward	GATCGGGGTGCCTGTTTAC-CGAACTAATCGAAATTAGTTCGG-TAAACAGGCACTTTTTC	70.5	Not applicable	Not applicable
		Reverse	AATTGAAAAAGTGCCTGTTTAC-CGAACTAATTTCGATTAGTTCGG-TAAACAGGCACCCC	69.8		

	KDM6A#3	Forward	GATCGGGGACTATGAGTCTAGTTTAAA-GCGAACTTTAAACTAGACTCATAGTCTTTTTC	66.2	Not applicable	Not applicable
		Reverse	AATTGAAAAAGACTATGAGTCTAG-TTTAAAGTTCGCTTTAAACTA-GACTCATAGTCCCC	65.4		
	KDM6A#6	Forward	GATCGGGAAGACCACTCAGA-TAGTGAATCGAAATTCACTATCTGAG-TGGTCTTTTTTTC	68.1	Not applicable	Not applicable
		Reverse	AATTGAAAAAAAGACCACTCAGA-TAGTGAATTTCGATTCACTATCTGAG-TGGTCTTCCC	67.4		
	KDM6B	Forward	GATCGGGAGTCCCACTCACCTCTATTTACGAA-TAAATAGAGGTGAGTGGGACTTTTTTC	69.3	Not applicable	Not applicable
		Reverse	AATTGAAAAAAGTCCCACTCACCTC-TATTTATTCGTAAATAGAGGTGAGTGGGACTCCC	68.5		
	YY1#1	Forward	GATCGGGCGATGGTTGTAATAA-GAAGTTCGAAAACTTCTTATTACAAC-CATCGTTTTTC	67.6	Not applicable	Not applicable
		Reverse	AATTGAAAAACGATGGTTGTAATAA-GAAGTTTTCGAACTTCTTATTACAACCATCGCCC	66.8		
	YY1#2	Forward	GATCGGGGCCTCTCCTTT-GTATATTATTCGAAAATAATATACAAAGGA-GAGGCTTTTTC	66.6	Not applicable	Not applicable
		Reverse	AATTGAAAAAGCCTCTCCTTT-GTATATTATTTTCGAATAATATACAAAGGA-GAGGCCCC	65.9		

	C-FOS#1	Forward	GATCGGGGCGGAGACAGACCAACTAGAAC-GAATTCTAGTTGGTCTGTCTCCGCTTTTTC	72.8	Not applicable	Not applicable
		Reverse	AATTGAAAAAGCGGAGACAGACCAACTA-GAATTCGTTCTAGTTGGTCTGTCTCCGCCCC	72.1		
	C-FOS#2	Forward	GATCGGGTCTGCTTTGCAGACCGAGAT-TCGAAAATCTCGGTCTGCAAAGCAGATTTTTC	72.3	Not applicable	Not applicable
		Reverse	AATTGAAAAATCTGCTTTGCAGACCGAGAT-TTTCGAATCTCGGTCTGCAAAGCAGACCC	71.7		
	TRKA#1	Forward	GATCGGGCATCGAGAACCCACAA-TACTTCGAAAAGTATTGTGGGTTCTCGATGTTTTTC	70.5	Not applicable	Not applicable
		Reverse	AATTGAAAAACATCGAGAACCCACAA-TACTTTTCGAAGTATTGTGGGTTCTCGATGCCC	69.7		
	TRKA#2	Forward	GATCGGGCCTGCTGGCTTGGCTGA-TACTCGAAAGTATCAGCCAAGCCAG-CAGGTTTTTC	75.3	Not applicable	Not applicable
		Reverse	AATTGAAAAACCTGCTGGCTTGGCTGA-TACTTTCGAGTATCAGCCAAGCCAGCAGGCCC	74.6		
**Q-PCR**	actin	Forward	AAGACCTGTACGCCAACACAG	53.8	88	101
		Reverse	AGGGCAGTGATCTCCTTCT	55.2		
	KDM1B	Forward	CTCTCCTGTGGGGAACATTTC	55.1	126	102
		Reverse	GACTAGGTTCGGTTTTGCCATT	55.9		
	KDM4B	Forward	AGACGTATGATGACATCGACGA	55.9	227	96
		Reverse	CGTAGATCGGGGAGACAAAGG	56.8		
	KDM5B	Forward	CAATGCTGTGGACCTGTATGT	55.1	229	98
		Reverse	TACGGAGGGTATAGTCCCTGG	56.3		

	KDM6A	Forward	GGACATGCTGTGTCACATCCT	57.2	189	101
		Reverse	CTCCTGTTGGTCTCATTTGGTG	55.9		
	KDM6B	Forward	CGCTGCCTCACCCATATCC	57.5	186	103
		Reverse	ATCCGCGACCTCTGAACTCT	57.6		
	NTRK1	Forward	CCATTTCACTCCTCGGCTCA	56.7	194	102
		Reverse	TGCAGCTTCTGTTCAGGCACT	59.3		
**ChIP**	NTRK1-A	Forward	CTTGCCCCTGTTTGATGATTG	54.6	83	103
		Reverse	CCTGAGGACCGAAGAATCAAACT	56.8		
	NTRK1-B	Forward	TGTTAGCCCTTGGTCCATGAA	56	85	102
		Reverse	TTGCAGGAGCACAAGAAAAGC	56.8		
	NTRK1-C	Forward	TGAGATTTCTCCCCTCCTAAAGGT	56.8	120	97
		Reverse	CAAGTAGGCAGCTGGAATTCAGA	57.2		
	NTRK1-D	Forward	GCTGGGCTGGTCTCTTAAGGA	58.3	104	98
		Reverse	CGGGCATGCCTCCCTTAC	57.6		
	NTRK1-E	Forward	AGAAGAGAGGGCTGGGAGATAAAG	57.5	86	101
		Reverse	CTGTCCCTAAGGCCTCGAATG	57		
	NTRK1-F	Forward	GAAGGACTGAGACGGGAATGTG	57.4	100	96
		Reverse	ATGACCAGGGTGCAGTGCAT	59		
	NTRK1-G	Forward	CTGCCCTAGCAAGCTTTATGGT	57.3	87	95
		Reverse	CCCCTTCGATCTCCCTGAAG	56.2		
	NTRK1-H	Forward	CCGATGAGAGTAGCCACAGGTAA	58	96	102
		Reverse	GGTGGAGCCAGCTTTTTGG	56.5		
	Gene_desert	Forward	CATCCCTGGACTGATTGTCA	53.9	200	97
		Reverse	GGTTGGCCAGGTACATGTTT	55.3		
**Point mutant**	KDM6A[ED]	Forward	AACACCAGGTGCGCAGGCGAA-TAACAACTTCTGTTCAGTT	69.5	Not applicable	Not applicable

		Reverse	AGTTGTTATTCGCCTGCGCAC-CTGGTGTTCTGCTCCCTGG	72.8		
**gRNA**	KDM6A-g1	Forward	CACCGGTTGCACAGGTACGATCTAC	61.6	Not applicable	Not applicable
		Reverse	AAACGTAGATCGTACCTGTGCAACC	59.6		
	KDM6A-g2	Forward	CACCGGAGTGAGTGCGTTTCGCTGC	66.7	Not applicable	Not applicable
		Reverse	AAACGCAGCGAAACGCACTCACTCC	64.8		
**Identification of mutant**	KDM6A-I	Forward	ACATCTTCATATTCATCTGG	47	Not applicable	Not applicable
		Reverse	TTGCTAAGTTATCTATGTCG	47.2		
